# Supplementary material for: Design of CellProfiler-Based Pipelines Enabling the Attribution of Molecular Stress Markers to Specific Tissue and Subcellular Compartments of the Colonic Mucosa
Source: Cell Mol Gastroenterol Hepatol. 2025 Dec 5;20(6):101680. doi: 10.1016/j.jcmgh.2025.101680 (PMC13094656; doi:10.1016/j.jcmgh.2025.101680)
Supplement: Supplementary Tables 1 and 2 [file mmc1.pdf]

Key,Value
CellProfiler_Version,4.2.7
ChannelType_DAPI,Grayscale
ChannelType_EPCAM,Grayscale
ChannelType_G3BP1,Grayscale
Metadata_Tags,"[""ImageNumber""]"
Pipeline_Pipeline,"CellProfiler Pipeline: http://www.cellprofiler.org
Version:5
DateRevision:427
GitHash:
ModuleCount:21
HasImagePlaneDetails:False

Images:[module_num:1|svn_version:'Unknown'|variable_revision_number:2|show_window:False|notes:['To begin creating your project, use the Images module to compile a list of files and/or folders that you want to analyze. You can also specify a set of rules to include only the desired files in your selected folders.']|batch_state:array([], dtype=uint8)|enabled:True|wants_pause:False]

:
Filter images?:Images only
Select the rule criteria:and (extension does isimage) (directory doesnot containregexp ""[\\\\/]\\."")

Metadata:[module_num:2|svn_version:'Unknown'|variable_revision_number:6|show_window:False|notes:['The Metadata module optionally allows you to extract information describing your images (i.e, metadata) which will be stored along with your measurements. This information can be contained in the file name and/or location, or in an external file.']|batch_state:array([], dtype=uint8)|enabled:True|wants_pause:False]
Extract metadata?:No
Metadata data type:Text
Metadata types:{}
Extraction method count:1
Metadata extraction method:Extract from file/folder names
Metadata source:File name

Regular expression to extract from file name:^(?P<Plate>.*)_(?P<Well>[A-P][0-9]{2})_s(?P<Site>[0-9])_w(?P<ChannelNumber>[0-9])
Regular expression to extract from folder name:(?P<Date>[0-9]{4}_[0-9]{2}_[0-9]{2})$
Extract metadata from:All images
Select the filtering criteria:and (file does contain """")
Metadata file location:Elsewhere...|
Match file and image metadata:[]
Use case insensitive matching?:No
Metadata file name:None
Does cached metadata exist?:No

NamesAndTypes:[module_num:3|svn_version:'Unknown'|variable_revision_number:8|show_window:False|notes:['The NamesAndTypes module allows you to assign a meaningful name to each image by which other modules will refer to it.']|batch_state:array([], dtype=uint8)|enabled:True|wants_pause:False]
Assign a name to:Images matching rules
Select the image type:Grayscale image
Name to assign these images:DNA
Match metadata:[]
Image set matching method:Order
Set intensity range from:Image metadata
Assignments count:3
Single images count:0
Maximum intensity:255.0
Process as 3D?:No
Relative pixel spacing in X:1.0
Relative pixel spacing in Y:1.0
Relative pixel spacing in Z:1.0
Select the rule criteria:and (file does containregexp ""ch01"")
Name to assign these images:DAPI
Name to assign these objects:Cell
Select the image type:Grayscale image
Set intensity range from:Image metadata
Maximum intensity:255.0
Select the rule criteria:and (file does containregexp ""ch00"")

    Name to assign these images:EPCAM
    Name to assign these objects:Cell
    Select the image type:Grayscale image
    Set intensity range from:Image metadata
    Maximum intensity:255.0
    Select the rule criteria:and (file does containregexp ""ch02"")
    Name to assign these images:G3BP1
    Name to assign these objects:Cell
    Select the image type:Grayscale image
    Set intensity range from:Image metadata
    Maximum intensity:255.0

Groups:[module_num:4|svn_version:'Unknown'|variable_revision_number:2|show_window:False|notes:['The Groups module optionally allows you to split your list of images into image subsets (groups) which will be processed independently of each other. Examples of groupings include screening batches, microtiter plates, time-lapse movies, etc.']|batch_state:array([], dtype=uint8)|enabled:True|wants_pause:False]
    Do you want to group your images?:No
    grouping metadata count:1
    Metadata category:None

CorrectIlluminationApply:[module_num:5|svn_version:'Unknown'|variable_revision_number:5|show_window:False|notes:['Accurate segmentation of nucleus objects is crucial for subsequent steps and is highly dependent on the effectiveness of the staining process. Substracting EpCAM from the DAPI channel increases the distance between adjacnt nuclei, improving segmentation in the IdentifyPrimaryObjects module. ']|batch_state:array([], dtype=uint8)|enabled:True|wants_pause:False]
    Select the input image:DAPI
    Name the output image:CorrDAPI
    Select the illumination function:EPCAM
    Select how the illumination function is applied:Subtract
    Set output image values less than 0 equal to 0?:Yes
    Set output image values greater than 1 equal to 1?:Yes

IdentifyPrimaryObjects:[module_num:6|svn_version:'Unknown'|variable_revision_number:15|show_window:False|notes:['Over- or under segmentation is common, particuarly in complex tissues. To minimize this, it is recommended to acquire high-quality images. However, it should be noted that approx. 10% error rate per image can be expected.']|batch_state:array([], dtype=uint8)|enabled:True|wants_pause:False]
    Select the input image:CorrDAPI
    Name the primary objects to be identified:IdentifyNucleiObjects
    Typical diameter of objects, in pixel units (Min,Max):15,75
    Discard objects outside the diameter range?:Yes
    Discard objects touching the border of the image?:Yes
    Method to distinguish clumped objects:Shape
    Method to draw dividing lines between clumped objects:Shape
    Size of smoothing filter:10
    Suppress local maxima that are closer than this minimum allowed distance:5
    Speed up by using lower-resolution image to find local maxima?:Yes
    Fill holes in identified objects?:After both thresholding and declumping
    Automatically calculate size of smoothing filter for declumping?:Yes
    Automatically calculate minimum allowed distance between local maxima?:Yes
    Handling of objects if excessive number of objects identified:Continue
    Maximum number of objects:500
    Use advanced settings?:Yes
    Threshold setting version:12
    Threshold strategy:Global
    Thresholding method:Minimum Cross-Entropy
    Threshold smoothing scale:1.3488
    Threshold correction factor:1.0
    Lower and upper bounds on threshold:0.1,1.0
    Manual threshold:0.0
    Select the measurement to threshold with:None
    Two-class or three-class thresholding?:Two classes
    Log transform before thresholding?:No
    Assign pixels in the middle intensity class to the foreground or the background?:Foreground
    Size of adaptive window:50
    Lower outlier fraction:0.05

    Upper outlier fraction:0.05
    Averaging method:Mean
    Variance method:Standard deviation
    # of deviations:2.0
    Thresholding method:Minimum Cross-Entropy

IdentifyPrimaryObjects:[module_num:7|svn_version:'Unknown'|variable_revision_number:15|show_window:False|notes:['The IdentifyPrimaryObject module was chosen for segmenting the intestinal region due to its flexibility compared to creating a mask. EpCAm was occasionally found in the lamina propria. While there are alternative methods for filtering out specific regions in CellProfiler, this option yielded the best results four our images and region. ']|batch_state:array([], dtype=uint8)|enabled:True|wants_pause:False]
    Select the input image:EPCAM
    Name the primary objects to be identified:IdentifyIntestine
    Typical diameter of objects, in pixel units (Min,Max):40,900
    Discard objects outside the diameter range?:Yes
    Discard objects touching the border of the image?:No
    Method to distinguish clumped objects:None
    Method to draw dividing lines between clumped objects:Shape
    Size of smoothing filter:10
    Suppress local maxima that are closer than this minimum allowed distance:7.0
    Speed up by using lower-resolution image to find local maxima?:Yes
    Fill holes in identified objects?:After declumping only
    Automatically calculate size of smoothing filter for declumping?:Yes
    Automatically calculate minimum allowed distance between local maxima?:Yes
    Handling of objects if excessive number of objects identified:Continue
    Maximum number of objects:500
    Use advanced settings?:Yes
    Threshold setting version:12
    Threshold strategy:Global
    Thresholding method:Minimum Cross-Entropy
    Threshold smoothing scale:1.3488
    Threshold correction factor:0.70
    Lower and upper bounds on threshold:0.01,1.0
    Manual threshold:0.0

Select the measurement to threshold with:None
Two-class or three-class thresholding?:Two classes
Log transform before thresholding?:No
Assign pixels in the middle intensity class to the foreground or the background?:Foreground
Size of adaptive window:50
Lower outlier fraction:0.05
Upper outlier fraction:0.05
Averaging method:Mean
Variance method:Standard deviation
# of deviations:2.0
Thresholding method:Minimum Cross-Entropy

MaskObjects:[module_num:8|svn_version:'Unknown'|variable_revision_number:3|show_window:False|notes:['First, the previous specified intestinal region was used for masking. Second, objects that are outside this region are removed, depending on overlap. ']|batch_state:array([], dtype=uint8)|enabled:True|wants_pause:False]
Select objects to be masked:IdentifyNucleiObjects
Name the masked objects:Nuclei
Mask using a region defined by other objects or by binary image?:Objects
Select the masking object:IdentifyIntestine
Select the masking image:None
Handling of objects that are partially masked:Remove
Fraction of object that must overlap:0.5
Numbering of resulting objects:Renumber
Invert the mask?:No

IdentifySecondaryObjects:[module_num:9|svn_version:'Unknown'|variable_revision_number:10|show_window:True|notes:['The nuclei objects are now used to identify whole cells. This is not a straightforward process for intestinal epithelial cells, as Goblet or Paneth cells have a large cytoplasm due to their secretory tasks. Unfortunately, we have not found a way to segment this region in secretory cells. It would be interestingto explore markers for specific segmentation of, for example, goblet cells. ', '', '']|batch_state:array([], dtype=uint8)|enabled:True|wants_pause:False]
Select the input objects:Nuclei
Name the objects to be identified:IdentifyCellObjects
Select the method to identify the secondary objects:Distance - N

Select the input image:EPCAM
Number of pixels by which to expand the primary objects:10
Regularization factor:0.05
Discard secondary objects touching the border of the image?:No
Discard the associated primary objects?:No
Name the new primary objects:FilteredNuclei
Fill holes in identified objects?:Yes
Threshold setting version:12
Threshold strategy:Global
Thresholding method:Minimum Cross-Entropy
Threshold smoothing scale:1.0
Threshold correction factor:1.0
Lower and upper bounds on threshold:0.01,1.0
Manual threshold:0.0
Select the measurement to threshold with:None
Two-class or three-class thresholding?:Two classes
Log transform before thresholding?:No
Assign pixels in the middle intensity class to the foreground or the background?:Foreground
Size of adaptive window:50
Lower outlier fraction:0.05
Upper outlier fraction:0.05
Averaging method:Mean
Variance method:Standard deviation
# of deviations:2.0
Thresholding method:Minimum Cross-Entropy

IdentifyTertiaryObjects:[module_num:10|svn_version:'Unknown'|variable_revision_number:3|show_window:True|notes:['Here, cytoplasm of cells are defined. ']|batch_state:array([], dtype=uint8)|enabled:True|wants_pause:False]
Select the larger identified objects:IdentifyCellObjects
Select the smaller identified objects:Nuclei
Name the tertiary objects to be identified:Cytoplasm
Shrink smaller object prior to subtraction?:Yes

EnhanceOrSuppressFeatures:[module_num:11|svn_version:'Unknown'|variable_revision_number:7|show_window:True|notes:['Enhancing features such as speckles can increase the contrast between the objects of interest and the backgroud, making it easier to distinguish specific features within the image. Also, algorithms can detect more accurately, segment, and classify objects. ', '', '']|batch_state:array([], dtype=uint8)|enabled:True|wants_pause:False]
    Select the input image:G3BP1
    Name the output image:EnhanceOrSuppressFeatures
    Select the operation:Enhance
    Feature size:10
    Feature type:Speckles
    Range of hole sizes:1,10
    Smoothing scale:2.0
    Shear angle:0.0
    Decay:0.95
    Enhancement method:Line structures
    Speed and accuracy:Fast
    Rescale result image:Yes

IdentifyPrimaryObjects:[module_num:12|svn_version:'Unknown'|variable_revision_number:15|show_window:True|notes:[]|batch_state:array([], dtype=uint8)|enabled:True|wants_pause:False]
    Select the input image:EnhanceOrSuppressFeatures
    Name the primary objects to be identified:IdentifyG3BP1Objects
    Typical diameter of objects, in pixel units (Min,Max):2,30
    Discard objects outside the diameter range?:Yes
    Discard objects touching the border of the image?:Yes
    Method to distinguish clumped objects:Intensity
    Method to draw dividing lines between clumped objects:Intensity
    Size of smoothing filter:10
    Suppress local maxima that are closer than this minimum allowed distance:7.0
    Speed up by using lower-resolution image to find local maxima?:Yes
    Fill holes in identified objects?:After both thresholding and declumping
    Automatically calculate size of smoothing filter for declumping?:Yes
    Automatically calculate minimum allowed distance between local maxima?:Yes
    Handling of objects if excessive number of objects identified:Continue

Maximum number of objects:500
Use advanced settings?:Yes
Threshold setting version:12
Threshold strategy:Global
Thresholding method:Minimum Cross-Entropy
Threshold smoothing scale:2
Threshold correction factor:1.0
Lower and upper bounds on threshold:0.04,1.0
Manual threshold:0.0
Select the measurement to threshold with:None
Two-class or three-class thresholding?:Two classes
Log transform before thresholding?:No
Assign pixels in the middle intensity class to the foreground or the background?:Foreground
Size of adaptive window:50
Lower outlier fraction:0.05
Upper outlier fraction:0.05
Averaging method:Mean
Variance method:Standard deviation
# of deviations:2.0
Thresholding method:Minimum Cross-Entropy

RelateObjects:[module_num:13|svn_version:'Unknown'|variable_revision_number:5|show_window:False|notes:['Here, we relate previous identifiy G3BP1 spots with cells, cytoplasm or nucleus (in seperate steps) to compare intensity, respectively. ', '', 'Note: The output later is extensive, so take the time to labbel objects appropriate, to retrieve desired measurements. ']|batch_state:array([], dtype=uint8)|enabled:True|wants_pause:False]
Parent objects:IdentifyCellObjects
Child objects:IdentifyG3BP1Objects
Calculate child-parent distances?:None
Calculate per-parent means for all child measurements?:No
Calculate distances to other parents?:No
Do you want to save the children with parents as a new object set?:Yes
Name the output object:RelateG3BP1ToCellObjects
Parent name:None

RelateObjects:[module_num:14|svn_version:'Unknown'|variable_revision_number:5|show_window:False|notes:[]|batch_state:array([], dtype=uint8)|enabled:True|wants_pause:False]
    Parent objects:Cytoplasm
    Child objects:IdentifyG3BP1Objects
    Calculate child-parent distances?:None
    Calculate per-parent means for all child measurements?:Yes
    Calculate distances to other parents?:No
    Do you want to save the children with parents as a new object set?:Yes
    Name the output object:RelateG3BP1ToCytoplasmObjects
    Parent name:None

RelateObjects:[module_num:15|svn_version:'Unknown'|variable_revision_number:5|show_window:False|notes:[]|batch_state:array([], dtype=uint8)|enabled:True|wants_pause:False]
    Parent objects:Nuclei
    Child objects:IdentifyG3BP1Objects
    Calculate child-parent distances?:None
    Calculate per-parent means for all child measurements?:Yes
    Calculate distances to other parents?:No
    Do you want to save the children with parents as a new object set?:Yes
    Name the output object:RelateG3BP1ToNucleusObjects
    Parent name:None

FilterObjects:[module_num:16|svn_version:'Unknown'|variable_revision_number:10|show_window:True|notes:['To compare distribution on how many cells are positive or negative for G3BP1 staining, previous identified cell objects were filtered for G3BP1.']|batch_state:array([], dtype=uint8)|enabled:True|wants_pause:False]
    Select the objects to filter:IdentifyCellObjects
    Name the output objects:G3BP1negativeCells
    Select the filtering mode:Measurements
    Select the filtering method:Limits
    Select the objects that contain the filtered objects:None
    Select the location of the rules or classifier file:Elsewhere...|
    Rules or classifier file name:rules.txt

Class number:1
Measurement count:1
Additional object count:0
Assign overlapping child to:Both parents
Keep removed objects as a separate set?:Yes
Name the objects removed by the filter:G3BP1positiveCells
Select the measurement to filter by:Children_IdentifyG3BP1Objects_Count
Filter using a minimum measurement value?:No
Minimum value:1
Filter using a maximum measurement value?:Yes
Maximum value:1.0
Allow fuzzy feature matching?:No

OverlayOutlines:[module_num:17|svn_version:'Unknown'|variable_revision_number:4|show_window:True|notes:['This module gives an overview on all identified objects and their relationsship.', 'Optional: This image can then be saved using the SaveImage module. ']|batch_state:array([], dtype=uint8)|enabled:True|wants_pause:False]
Display outlines on a blank image?:No
Select image on which to display outlines:CorrDAPI
Name the output image:OrigOverlay
Outline display mode:Color
Select method to determine brightness of outlines:Max of image
How to outline:Inner
Select outline color:#00DFDF
Select objects to display:Nuclei
Select outline color:#C94BAA
Select objects to display:G3BP1positiveCells
Select outline color:#F4F400
Select objects to display:RelateG3BP1ToCellObjects
Select outline color:white
Select objects to display:G3BP1negativeCells

MeasureObjectSizeShape:[module_num:18|svn_version:'Unknown'|variable_revision_number:3|show_window:False|notes:['To compare G3BP1 spots based on cell size, size of G3BP1 spots, cells, nucleus and cytoplasm were selected.']|batch_state:array([], dtype=uint8)|enabled:True|wants_pause:False]
    Select object sets to measure:IdentifyG3BP1Objects, RelateG3BP1ToCellObjects, RelateG3BP1ToCytoplasmObjects, RelateG3BP1ToNucleusObjects
    Calculate the Zernike features?:No
    Calculate the advanced features?:No

MeasureObjectIntensity:[module_num:19|svn_version:'Unknown'|variable_revision_number:4|show_window:False|notes:['To compare mean or max intensity of G3BP1 in cell, cytoplasm, or nuclei objects, G3BP1 as image was selected andcCytoplams, G3BP1-positive cells, and nuclei as objects.
']|batch_state:array([], dtype=uint8)|enabled:True|wants_pause:False]
    Select images to measure:G3BP1
    Select objects to measure:Cytoplasm, IdentifyCellObjects, Nuclei

MeasureObjectIntensityDistribution:[module_num:20|svn_version:'Unknown'|variable_revision_number:6|show_window:False|notes:[]|batch_state:array([], dtype=uint8)|enabled:True|wants_pause:False]
    Select images to measure:G3BP1
    Hidden:1
    Hidden:1
    Hidden:0
    Calculate intensity Zernikes?:None
    Maximum zernike moment:9
    Select objects to measure:G3BP1positiveCells
    Object to use as center?:Edges of other objects
    Select objects to use as centers:Nuclei
    Scale the bins?:Yes
    Number of bins:3
    Maximum radius:100

ExportToSpreadsheet:[module_num:21|svn_version:'Unknown'|variable_revision_number:13|show_window:True|notes:['Here, data are selected for export. ']|batch_state:array([], dtype=uint8)|enabled:True|wants_pause:False]
    Select the column delimiter:Comma ("","")
    Add image metadata columns to your object data file?:Yes
    Add image file and folder names to your object data file?:No

Select the measurements to export:Yes
Calculate the per-image mean values for object measurements?:Yes
Calculate the per-image median values for object measurements?:No
Calculate the per-image standard deviation values for object measurements?:No
Output file location:Default Input Folder sub-folder|Desktop\\Submit\\Paper_supplementary\\EpitheliaCellProfiler
Create a GenePattern GCT file?:No
Select source of sample row name:Metadata
Select the image to use as the identifier:None
Select the metadata to use as the identifier:None
Export all measurement types?:Yes

measurements:RelateG3BP1ToCellObjects|AreaShape_BoundingBoxMinimum_X,RelateG3BP1ToCellObjects|AreaShape_BoundingBoxMinimum_Y,RelateG3BP1ToCellObjects|AreaShape_Eccentricity,RelateG3BP1ToCellObjects|AreaShape_Solidity,RelateG3BP1ToCellObjects|AreaShape_EulerNumber,RelateG3BP1ToCellObjects|AreaShape_ConvexArea,RelateG3BP1ToCellObjects|AreaShape_MaximumRadius,RelateG3BP1ToCellObjects|AreaShape_MinFeretDiameter,RelateG3BP1ToCellObjects|AreaShape_MeanRadius,RelateG3BP1ToCellObjects|AreaShape_Perimeter,RelateG3BP1ToCellObjects|AreaShape_Area,RelateG3BP1ToCellObjects|AreaShape_Orientation,RelateG3BP1ToCellObjects|AreaShape_BoundingBoxMaximum_Y,RelateG3BP1ToCellObjects|AreaShape_BoundingBoxMaximum_X,RelateG3BP1ToCellObjects|AreaShape_MedianRadius,RelateG3BP1ToCellObjects|AreaShape_MaxFeretDiameter,RelateG3BP1ToCellObjects|AreaShape_Compactness,RelateG3BP1ToCellObjects|AreaShape_Center_X,RelateG3BP1ToCellObjects|AreaShape_Center_Y,RelateG3BP1ToCellObjects|AreaShape_MajorAxisLength,RelateG3BP1ToCellObjects|AreaShape_BoundingBoxArea,RelateG3BP1ToCellObjects|AreaShape_MinorAxisLength,RelateG3BP1ToCellObjects|AreaShape_EquivalentDiameter,RelateG3BP1ToCellObjects|AreaShape_FormFactor,RelateG3BP1ToCellObjects|AreaShape_Extent,IdentifyG3BP1Objects|Parent_Cytoplasm,IdentifyG3BP1Objects|Parent_Nuclei,IdentifyG3BP1Objects|Parent_IdentifyCellObjects,IdentifyG3BP1Objects|AreaShape_Eccentricity,IdentifyG3BP1Objects|AreaShape_MaximumRadius,IdentifyG3BP1Objects|AreaShape_MaxFeretDiameter,IdentifyG3BP1Objects|AreaShape_Area,IdentifyG3BP1Objects|AreaShape_BoundingBoxMaximum_X,IdentifyG3BP1Objects|AreaShape_BoundingBoxMaximum_Y,IdentifyG3BP1Objects|AreaShape_MinFeretDiameter,IdentifyG3BP1Objects|AreaShape_MajorAxisLength,IdentifyG3BP1Objects|AreaShape_MinorAxisLength,IdentifyG3BP1Objects|AreaShape_Compactness,IdentifyG3BP1Objects|AreaShape_Center_X,IdentifyG3BP1Objects|AreaShape_Center_Y,IdentifyG3BP1Objects|AreaShape_Solidity,IdentifyG3BP1Objects|AreaShape_MeanRadius,IdentifyG3BP1Objects|AreaShape_BoundingBoxArea,IdentifyG3BP1Objects|AreaShape_FormFactor,IdentifyG3BP1Objects|AreaShape_Orientation,IdentifyG3BP1Objects|AreaShape_EquivalentDiameter,IdentifyG3BP1Objects|AreaShape_BoundingBoxMinimum_Y,IdentifyG3BP1Objects|AreaShape_BoundingBoxMinimum_X,IdentifyG3BP1Objects|AreaShape_MedianRadius,IdentifyG3BP1Objects|AreaShape_Extent,IdentifyG3BP1Objects|AreaShape_EulerNumber,IdentifyG3BP1Objects|AreaShape_Perimeter,IdentifyG3BP1Objects|AreaShape_ConvexArea,IdentifyG3BP1Objects|Children_RelateG3BP1ToCellObjects_Count,IdentifyG3BP1Objects|Children_RelateG3BP1ToCytoplasmObjects_Count,IdentifyG3BP1Objects|Children_RelateG3BP1ToNucleusObjects_Count,IdentifyG3BP1Objects|Number_Object_Number,RelateG3BP1ToNucleusObjects|AreaShape_FormFactor,RelateG3BP1ToNucleusObjects|AreaShape_MaxFeretDiameter,RelateG3BP1ToNucleusObjects|AreaShape_MajorAxisLength,RelateG3BP1ToNucleusObjects|AreaShape_EquivalentDiameter,RelateG3BP1ToNucleusObjects|AreaShape_Eccentricity,RelateG3BP1ToNucleusObjects|AreaShape_BoundingBoxMinimum_Y,RelateG3BP1ToNucleusObjects|AreaShape_BoundingBoxMinimum_X,RelateG3BP1ToNucleusObjects|AreaShape_MaximumRadius,RelateG3BP1ToNucleusObjects|AreaShape_BoundingBoxMaximum_Y,RelateG3BP1ToNucleusObjects|AreaShape_BoundingBoxMaximum_X,RelateG3BP1ToNucleusObjects|AreaShape_Compactness,RelateG3BP1ToNucleusObjects|AreaShape_MinFeretDiameter,RelateG3BP1ToNucleusObjects|AreaShape_Extent,RelateG3BP1ToNucleusObjects|AreaShape_ConvexArea,RelateG3BP1ToNucleusObjects|AreaShape_Perimeter,RelateG3BP1ToNucleusObjects|AreaShape_BoundingBoxArea,RelateG3BP1ToNucleusObjects|AreaShape_Area,RelateG3BP1ToNucleusObjects|AreaShape_Center_Y,RelateG3BP1ToNucleusObjects|AreaShape_Center_X,RelateG3BP1ToNucleusObjects|AreaShape_MedianRadius,RelateG3BP1ToNucleusObjects|AreaShape_EulerNumber,RelateG3BP1ToNucleusObjects|AreaShape_MeanRadius,RelateG3BP1To

Representation of Nan/Inf:Null

Add a prefix to file names?:Yes

Filename prefix:25032025

Overwrite existing files without warning?:Yes

Data to export:Do not use
Combine these object measurements with those of the previous object?:No
File name:DATA.csv
Use the object name for the file name?:Yes

Key,Value
CellProfiler_Version,4.2.7
ChannelType_DAPI,Grayscale
ChannelType_EpCAM,Grayscale
ChannelType_G3BP1,Grayscale
Metadata_Tags,"[""ImageNumber""]"
Pipeline_Pipeline,"CellProfiler Pipeline: http://www.cellprofiler.org
Version:5
DateRevision:427
GitHash:
ModuleCount:20
HasImagePlaneDetails:False

Images:[module_num:1|svn_version:'Unknown'|variable_revision_number:2|show_window:False|notes:['To begin creating your project, use the Images module to compile a list of files and/or folders that you want to analyze. You can also specify a set of rules to include only the desired files in your selected folders.', '', '']|batch_state:array([], dtype=uint8)|enabled:True|wants_pause:False]
:
Filter images?:Custom
Select the rule criteria:or (extension does isimage) (file does contain ""overlay"")

Metadata:[module_num:2|svn_version:'Unknown'|variable_revision_number:6|show_window:False|notes:['The Metadata module optionally allows you to extract information describing your images (i.e, metadata) which will be stored along with your measurements. This information can be contained in the file name and/or location, or in an external file.']|batch_state:array([],
Extract metadata?:No
Metadata data type:Text
Metadata types:{}
Extraction method count:1
Metadata extraction method:Extract from file/folder names
Metadata source:File name
Regular expression to extract from file name:^(?P<Plate>.*)_(?P<Well>[A-P][0-9]{2})_s(?P<Site>[0-9])_w(?P<ChannelNumber>[0-9])
Regular expression to extract from folder name:(?P<Date>[0-9]{4}_[0-9]{2}_[0-9]{2})$
Extract metadata from:Images matching a rule
Select the filtering criteria:and (file does contain ""5103"")
Metadata file location:Elsewhere...|
Match file and image metadata:[]
Use case insensitive matching?:No
Metadata file name:None
Does cached metadata exist?:No

NamesAndTypes:[module_num:3|svn_version:'Unknown'|variable_revision_number:8|show_window:False|notes:['The NamesAndTypes module allows you to assign a meaningful name to each image by which other modules will refer to it.', '', '']|batch_state:array([], dtype=uint8)|enabled:True|wants_pause:False]
Assign a name to:Images matching rules
Select the image type:Color image
Name to assign these images:Image
Match metadata:[]
Image set matching method:Order

Set intensity range from:Image metadata
Assignments count:3
Single images count:0
Maximum intensity:255.0
Process as 3D?:No
Relative pixel spacing in X:1.0
Relative pixel spacing in Y:1.0
Relative pixel spacing in Z:1.0
Select the rule criteria:and (file does containregexp ""ch00"")
Name to assign these images:EpCAM
Name to assign these objects:Cell
Select the image type:Grayscale image
Set intensity range from:Image metadata
Maximum intensity:255.0
Select the rule criteria:and (file does containregexp ""ch01"")
Name to assign these images:DAPI
Name to assign these objects:Cell
Select the image type:Grayscale image
Set intensity range from:Image metadata
Maximum intensity:255.0
Select the rule criteria:and (file does containregexp ""ch02"")
Name to assign these images:G3BP1
Name to assign these objects:Cell
Select the image type:Grayscale image
Set intensity range from:Image metadata
Maximum intensity:255.0

Groups:[module_num:4|svn_version:'Unknown'|variable_revision_number:2|show_window:False|notes:['The Groups module optionally allows you to split your list of images into image subsets (groups) which will be processed independently of each other. Examples of groupings include screening batches, microtiter plates, time-lapse movies, etc.']|batch_state:array([],
Do you want to group your images?:No
grouping metadata count:1
Metadata category:None

CorrectIlluminationApply:[module_num:5|svn_version:'Unknown'|variable_revision_number:5|show_window:False|notes:['Accurate segmentation of nucleus objects is crucial for subsequent steps and is highly dependent on the effectiveness of the staining process. Substracting EpCAM signals from the DAPI channel aids in the segmentation of IEC and reduced the EpCAM signal that is mistakenely present in the lamina propria. This signal can be filtered out more easily in the later treshold settings additionally.
']|batch_state:array([], dtype=uint8)|enabled:True|wants_pause:False]
Select the input image:DAPI
Name the output image:CorrBlue
Select the illumination function:EpCAM
Select how the illumination function is applied:Subtract
Set output image values less than 0 equal to 0?:Yes
Set output image values greater than 1 equal to 1?:Yes

IdentifyPrimaryObjects:[module_num:6|svn_version:'Unknown'|variable_revision_number:15|show_window:True|notes:['Since potential immune cells and epithelial cells differ in size and morphology, it was necessary to seperate them into 2 different pipelines (LaminaCellProfiler and EpitheliaCellProfiler). Therefore, during segmentation of immune cell nuclei, objects in the intestinal area were not considered as the differences, as mentioned previolusy, were too significant. Objects inside the intestinal region are filtered out in the subsequent steps anyway. ', '', 'Over- or under segmentation is common, particuarly in complex tissues. To minimize this, it is recommended to acquire high-quality images. However, it should be noted that approx. 10% error rate per image can be expected. ']|batch_state:array([], dtype=uint8)|enabled:True|wants_pause:False]

Select the input image:CorrBlue
Name the primary objects to be identified:IdentifyNucleiObjects
Typical diameter of objects, in pixel units (Min,Max):12,70
Discard objects outside the diameter range?:Yes
Discard objects touching the border of the image?:Yes
Method to distinguish clumped objects:Shape
Method to draw dividing lines between clumped objects:Shape
Size of smoothing filter:10
Suppress local maxima that are closer than this minimum allowed distance:10
Speed up by using lower-resolution image to find local maxima?:Yes
Fill holes in identified objects?:After both thresholding and declumping
Automatically calculate size of smoothing filter for declumping?:No
Automatically calculate minimum allowed distance between local maxima?:No
Handling of objects if excessive number of objects identified:Continue
Maximum number of objects:500
Use advanced settings?:Yes
Threshold setting version:12
Threshold strategy:Global
Thresholding method:Minimum Cross-Entropy
Threshold smoothing scale:2
Threshold correction factor:1.0
Lower and upper bounds on threshold:0.12,1.0
Manual threshold:0.0
Select the measurement to threshold with:None
Two-class or three-class thresholding?:Two classes
Log transform before thresholding?:No
Assign pixels in the middle intensity class to the foreground or the background?:Foreground
Size of adaptive window:25
Lower outlier fraction:0.05
Upper outlier fraction:0.05
Averaging method:Mean
Variance method:Standard deviation
# of deviations:2.0
Thresholding method:Minimum Cross-Entropy

IdentifyPrimaryObjects:[module_num:7|svn_version:'Unknown'|variable_revision_number:15|show_window:False|notes:['Here, we define the intestinal region as an object based on the green (EpCAM) channel. ']|batch_state:array([], dtype=uint8)|enabled:True|wants_pause:False]

Select the input image:EpCAM
Name the primary objects to be identified:IdentifyIntestine
Typical diameter of objects, in pixel units (Min,Max):50,900

Discard objects outside the diameter range?:Yes
Discard objects touching the border of the image?:No
Method to distinguish clumped objects:None
Method to draw dividing lines between clumped objects:Intensity
Size of smoothing filter:10
Suppress local maxima that are closer than this minimum allowed distance:100
Speed up by using lower-resolution image to find local maxima?:Yes
Fill holes in identified objects?:After declumping only
Automatically calculate size of smoothing filter for declumping?:Yes
Automatically calculate minimum allowed distance between local maxima?:Yes
Handling of objects if excessive number of objects identified:Continue
Maximum number of objects:500
Use advanced settings?:Yes
Threshold setting version:12
Threshold strategy:Global
Thresholding method:Minimum Cross-Entropy
Threshold smoothing scale:1.3488
Threshold correction factor:1.0
Lower and upper bounds on threshold:0.02,1.0
Manual threshold:0.0
Select the measurement to threshold with:None
Two-class or three-class thresholding?:Two classes
Log transform before thresholding?:No
Assign pixels in the middle intensity class to the foreground or the background?:Foreground
Size of adaptive window:50
Lower outlier fraction:0.05
Upper outlier fraction:0.05
Averaging method:Mean
Variance method:Standard deviation
# of deviations:2.0
Thresholding method:Minimum Cross-Entropy

ExpandOrShrinkObjects:[module_num:8|svn_version:'Unknown'|variable_revision_number:2|show_window:False|notes:['Sometimes cells close to intestinal crypts overlap within EpCAM staining. By shrinking or expanding the EpCAM objects by few pixels, these cells can be kept or removed']|batch_state:array([], dtype=uint8)|enabled:True|wants_pause:False]
Select the input objects:IdentifyIntestine
Name the output objects:ShrunkenIntestine
Select the operation:Shrink objects by a specified number of pixels
Number of pixels by which to expand or shrink:1
Fill holes in objects so that all objects shrink to a single point?:Yes
Expand or shrink measurement:None

MaskObjects:[module_num:9|svn_version:'Unknown'|variable_revision_number:3|show_window:True|notes:['In this module, all DAPI-identified objects that can be found within the EpCAM-stained Mask are removed. ']|batch_state:array([], dtype=uint8)|enabled:True|wants_pause:False]
Select objects to be masked:IdentifyNucleiObjects
Name the masked objects:MaskedNuclei
Mask using a region defined by other objects or by binary image?:Objects
Select the masking object:ShrunkenIntestine

Select the masking image:None
Handling of objects that are partially masked:Remove
Fraction of object that must overlap:0.5
Numbering of resulting objects:Renumber
Invert the mask?:Yes

MeasureObjectSizeShape:[module_num:10|svn_version:'Unknown'|variable_revision_number:3|show_window:False|notes:['Size and nuclei form is measured in this step to filter out excessive cells (due to oversegmentation) and potentially myofibroblasts.']|batch_state:array([], dtype=uint8)|enabled:True|wants_pause:False]
Select object sets to measure:MaskedNuclei
Calculate the Zernike features?:No
Calculate the advanced features?:No

FilterObjects:[module_num:11|svn_version:'Unknown'|variable_revision_number:10|show_window:False|notes:['Myofibroblasts are contractile cells with a central role in the remodeling and the restoration of damaged gastrointestinal tissue. These subepithelial intestinal fibroblasts are present within the lamina propria and often surround the crypt epithelium. Immunologic makers exist to aid in the identification of these cellls, but none is absolutely specific. However, using ""MeasureObjectSizeShape"" myofibroblasts within the lamina propria can be filtered and seperataed from immune cells due to their characteristic shape and an eccentricity value over 0.9 if not sufficient seperated alreay in the previos masking step. ', 'Also, by filtering cells by their maximal area, we filtered out obvious oversegmented cells (two or three cells that were recognized as one). ']|batch_state:array([],
Select the objects to filter:MaskedNuclei
Name the output objects:Nuclei
Select the filtering mode:Measurements
Select the filtering method:Limits
Select the objects that contain the filtered objects:None
Select the location of the rules or classifier file:Elsewhere...|
Rules or classifier file name:rules.txt
Class number:1
Measurement count:2
Additional object count:0
Assign overlapping child to:Both parents
Keep removed objects as a separate set?:Yes
Name the objects removed by the filter:FilteredCells
Select the measurement to filter by:AreaShape_Eccentricity
Filter using a minimum measurement value?:No
Minimum value:0.0
Filter using a maximum measurement value?:Yes
Maximum value:0.97
Select the measurement to filter by:AreaShape_Area
Filter using a minimum measurement value?:No
Minimum value:0.0
Filter using a maximum measurement value?:Yes
Maximum value:1600
Allow fuzzy feature matching?:No

IdentifySecondaryObjects:[module_num:12|svn_version:'Unknown'|variable_revision_number:10|show_window:False|notes:['Here, cells outer edges are identified. In this study, no additional cell marker was used for immune cells, therefore, the Distance-N method was used to assign an outer edge, based on pixels that expand from the nucleus objects. ']|batch_state:array([],

    Select the input objects:Nuclei
    Name the objects to be identified:IdentifyCellObjects
    Select the method to identify the secondary objects:Distance - N
    Select the input image:CorrBlue
    Number of pixels by which to expand the primary objects:7
    Regularization factor:0.05
    Discard secondary objects touching the border of the image?:No
    Discard the associated primary objects?:No
    Name the new primary objects:FilteredNuclei
    Fill holes in identified objects?:Yes
    Threshold setting version:12
    Threshold strategy:Global
    Thresholding method:Minimum Cross-Entropy
    Threshold smoothing scale:0.0
    Threshold correction factor:1.0
    Lower and upper bounds on threshold:0.0,1.0
    Manual threshold:0.0
    Select the measurement to threshold with:None
    Two-class or three-class thresholding?:Two classes
    Log transform before thresholding?:No
    Assign pixels in the middle intensity class to the foreground or the background?:Foreground
    Size of adaptive window:50
    Lower outlier fraction:0.05
    Upper outlier fraction:0.05
    Averaging method:Mean
    Variance method:Standard deviation
    # of deviations:2.0
    Thresholding method:Minimum Cross-Entropy

EnhanceOrSuppressFeatures:[module_num:13|svn_version:'Unknown'|variable_revision_number:7|show_window:False|notes:['Enhancing features such as speckles can increase the contrast between the objects of interest and the backgroud, making it easier to distinguish specific features within the image. Also, algorithms can detect more accurately, segment, and classify objects. ']|batch_state:array([], dtype=uint8)|enabled:True|wants_pause:False]

    Select the input image:G3BP1
    Name the output image:EnhanceOrSuppressFeatures
    Select the operation:Enhance
    Feature size:10
    Feature type:Speckles
    Range of hole sizes:1,10
    Smoothing scale:2.0
    Shear angle:0.0
    Decay:0.95
    Enhancement method:Tubeness
    Speed and accuracy:Fast
    Rescale result image:No

IdentifyPrimaryObjects:[module_num:14|svn_version:'Unknown'|variable_revision_number:15|show_window:False|notes:['Here, we identify G3BP1 granules in the red (G3BP1) channel as object to related them in the next steps.']|batch_state:array([], dtype=uint8)|enabled:True|wants_pause:False]
    Select the input image:EnhanceOrSuppressFeatures
    Name the primary objects to be identified:IdentifySpotObjects
    Typical diameter of objects, in pixel units (Min,Max):2,15
    Discard objects outside the diameter range?:Yes
    Discard objects touching the border of the image?:Yes
    Method to distinguish clumped objects:Intensity
    Method to draw dividing lines between clumped objects:Intensity
    Size of smoothing filter:10
    Suppress local maxima that are closer than this minimum allowed distance:7.0
    Speed up by using lower-resolution image to find local maxima?:Yes
    Fill holes in identified objects?:After both thresholding and declumping
    Automatically calculate size of smoothing filter for declumping?:Yes
    Automatically calculate minimum allowed distance between local maxima?:Yes
    Handling of objects if excessive number of objects identified:Continue
    Maximum number of objects:500
    Use advanced settings?:Yes
    Threshold setting version:12
    Threshold strategy:Global
    Thresholding method:Otsu
    Threshold smoothing scale:2
    Threshold correction factor:1.0
    Lower and upper bounds on threshold:0.04,1.0
    Manual threshold:0.0
    Select the measurement to threshold with:None
    Two-class or three-class thresholding?:Two classes
    Log transform before thresholding?:No
    Assign pixels in the middle intensity class to the foreground or the background?:Foreground
    Size of adaptive window:50
    Lower outlier fraction:0.05
    Upper outlier fraction:0.05
    Averaging method:Mean
    Variance method:Standard deviation
    # of deviations:2.0
    Thresholding method:Sauvola

RelateObjects:[module_num:15|svn_version:'Unknown'|variable_revision_number:5|show_window:False|notes:['Here, we relate previous identifiy G3BP1 spots with cells, cytoplasm or nucleus (in seperate steps) to compare intensity, respectively. ', '', 'Note: The output later is extensive, so take the time to labbel objects appropriate, to retrieve desired measurements. ']|batch_state:array([], dtype=uint8)|enabled:True|wants_pause:False]
    Parent objects:IdentifyCellObjects
    Child objects:IdentifySpotObjects
    Calculate child-parent distances?:None
    Calculate per-parent means for all child measurements?:Yes
    Calculate distances to other parents?:No
    Do you want to save the children with parents as a new object set?:Yes

Name the output object:RelateG3BP1ToCellObjects
Parent name:None

FilterObjects:[module_num:16|svn_version:'Unknown'|variable_revision_number:10|show_window:False|notes:['To compare distribution on how many cells are positive or negative for G3BP1 staining, previous identified cell objects were filtered for G3BP1.']|batch_state:array([], dtype=uint8)|enabled:True|wants_pause:False]
Select the objects to filter:IdentifyCellObjects
Name the output objects:G3BP1negativeCells
Select the filtering mode:Measurements
Select the filtering method:Limits
Select the objects that contain the filtered objects:None
Select the location of the rules or classifier file:Elsewhere...|
Rules or classifier file name:rules.txt
Class number:1
Measurement count:1
Additional object count:0
Assign overlapping child to:Both parents
Keep removed objects as a separate set?:Yes
Name the objects removed by the filter:G3BP1positive
Select the measurement to filter by:Children_IdentifySpotObjects_Count
Filter using a minimum measurement value?:No
Minimum value:1
Filter using a maximum measurement value?:Yes
Maximum value:1.0
Allow fuzzy feature matching?:No

OverlayOutlines:[module_num:17|svn_version:'Unknown'|variable_revision_number:4|show_window:True|notes:['This module gives an overview on all identified objects and their relationssship.', 'Optional: This image can then be saved using the SaveImage module. ']|batch_state:array([], dtype=uint8)|enabled:True|wants_pause:False]
Display outlines on a blank image?:No
Select image on which to display outlines:DAPI
Name the output image:OrigOverlay1
Outline display mode:Color
Select method to determine brightness of outlines:Max of image
How to outline:Thick
Select outline color:#00CACA
Select objects to display:Nuclei
Select outline color:yellow
Select objects to display:RelateG3BP1ToCellObjects
Select outline color:white
Select objects to display:G3BP1negativeCells
Select outline color:#CA0065
Select objects to display:G3BP1positive

MeasureObjectIntensity:[module_num:18|svn_version:'Unknown'|variable_revision_number:4|show_window:False|notes:['Here we used MeasureOjectIntensity to measure pixel intensity of G3BP1 in G3BP1+ positive cells of healthy control and non-IBD']|batch_state:array([], dtype=uint8)|enabled:True|wants_pause:False]

    Select images to measure:G3BP1
    Select objects to measure:G3BP1positive, IdentifyCellObjects, IdentifySpotObjects

MeasureObjectSizeShape:[module_num:19|svn_version:'Unknown'|variable_revision_number:3|show_window:False|notes:['This module was used, to compare G3BP1 spot size and nuclei morphology between healthy control and ulcerative colitis patients in remission. ']|batch_state:array([], dtype=uint8)|enabled:True|wants_pause:False]
    Select object sets to measure:IdentifySpotObjects, Nuclei
    Calculate the Zernike features?:No
    Calculate the advanced features?:No

ExportToSpreadsheet:[module_num:20|svn_version:'Unknown'|variable_revision_number:13|show_window:False|notes:['Here, data are selected for export. ']|batch_state:array([], dtype=uint8)|enabled:True|wants_pause:False]
    Select the column delimiter:Comma ("","")
    Add image metadata columns to your object data file?:No
    Add image file and folder names to your object data file?:No
    Select the measurements to export:Yes
    Calculate the per-image mean values for object measurements?:No
    Calculate the per-image median values for object measurements?:No
    Calculate the per-image standard deviation values for object measurements?:No
    Output file location:Default Input Folder sub-folder|Desktop\\Submit\\Paper_supplementary\\PropiCellProfiler
    Create a GenePattern GCT file?:No
    Select source of sample row name:Metadata
    Select the image to use as the identifier:None
    Select the metadata to use as the identifier:None
    Export all measurement types?:Yes
    Press button to select measurements:Experiment|Run_Timestamp,Experiment|Modification_Timestamp,Experiment|Pipeline_Pipeline,Experiment|CellProfiler_Version
    Representation of Nan/Inf:Null
    Add a prefix to file names?:Yes
    Filename prefix:PropiCellProfiler_Output
    Overwrite existing files without warning?:Yes
    Data to export:Do not use
    Combine these object measurements with those of the previous object?:No
    File name:DATA.csv
    Use the object name for the file name?:Yes
