# Supplementary material for: Design of CellProfiler-Based Pipelines Enabling the Attribution of Molecular Stress Markers to Specific Tissue and Subcellular Compartments of the Colonic Mucosa
Source: Cell Mol Gastroenterol Hepatol. 2025 Dec 5;20(6):101680. doi: 10.1016/j.jcmgh.2025.101680 (PMC13094656; doi:10.1016/j.jcmgh.2025.101680)
Supplement: Extended PDF [file mmc2.pdf]

cmgh
CELLULAR AND MOLECULAR GASTROENTEROLOGY AND HEPATOLOGY

ORIGINAL RESEARCH

Design of CellProfiler-Based Pipelines Enabling the Attribution of Molecular Stress Markers to Specific Tissue and Subcellular Compartments of the Colonic Mucosa

Helena Hödlmayr,[1] Christina Watschinger,[1] Gerald K. Wallner,[1] Sabine Knipp,[2] Arndt Rohwedder,[2] Regina Prommer,[1] Rupert Langer,[3] and Alexander R. Moschen[1]

[1]Department of Internal Medicine 2 (Gastroenterology and Hepatology, Endocrinology, and Metabolism, Nephrology, Rheumatology), Faculty of Medicine, Johannes Kepler University Linz, Linz, Austria; [2]Core Facility Imaging, Faculty of Medicine, Johannes Kepler University Linz, Linz, Austria; and [3]Institute of Pathology und Molecular Pathology, Johannes Kepler University Linz, Linz, Austria

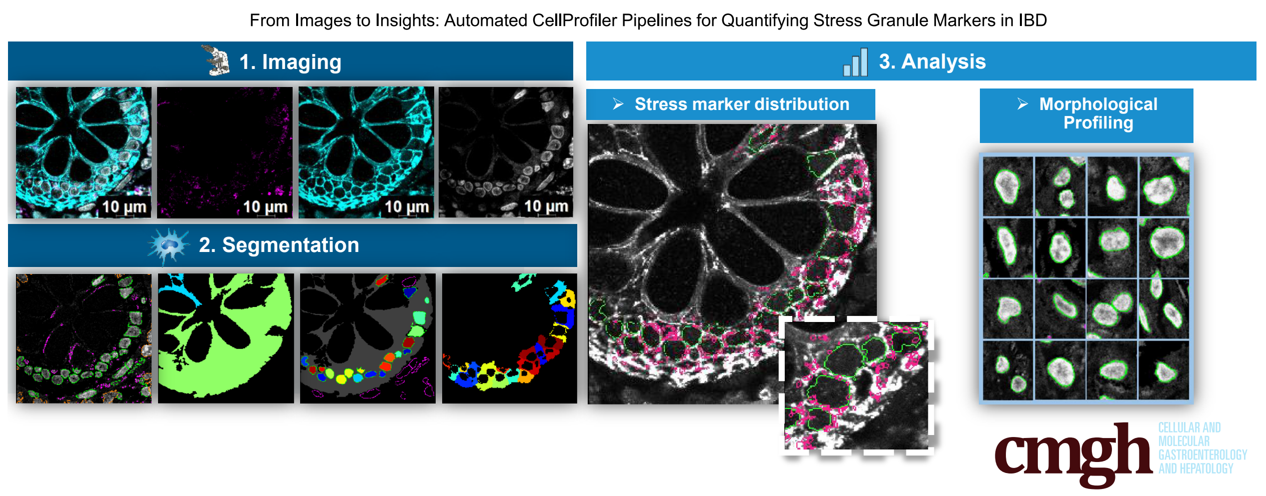

SUMMARY

We describe the development of CellProfiler-based pipelines for high-throughput analysis of confocal images from human colonic specimens, enabling quantitative assessment of nuclear morphology in inflammatory bowel disease and the distribution of stress granules in post inflammatory tissue.

BACKGROUND & AIMS: Stress granules (SGs) represent membrane-free cytoplasmic structures rapidly aggregating during cellular stress responses arguably useful as markers of molecular inflammation. To provide an automated, reproducible, and unbiased analytic workflow, we used the open-source software CellProfiler to quantify SGs in distinct cell types in inflammatory bowel disease.

METHODS: The EpiCellProfiler (ECP) and PropiCellProfiler (PCP) pipelines enable segmentation within intestinal epithelial cells and lamina propria cells, respectively. The SG marker Ras GTPase-activating protein-binding protein 1 (G3BP1) was quantified for fluorescence intensity, granule size, and morphology on tissue sections of patients with ulcerative colitis (UC) and Crohn's disease (CD) in deep remission.

RESULTS: Both pipelines detected elevated G3BP1 fluorescence intensities in inactive UC and CD. Additionally, SGs spot counts and spot sizes were increased in CD and UC compared with controls. The distribution of G3BP1 was homogenous in intestinal epithelial cells, without SG typical aggregations. In UC, PCP analysis revealed nuclear morphology alterations in terms of size, regularity, and compactness.

CONCLUSIONS: Herein, we provide a powerful, reproducible, versatile and open-source software tool to quantify remnant molecular inflammation in patients with CD and UC, enabling research to openly share, reproduce and compare results within the field of quantitative image analysis. Our pipeline separates and distinguishes between epithelial and lamina propria events and provides insights into the spatial distribution and dynamics of SGs, revealing their homogeneous distribution and persistent accumulation in patients with CD and UC, notably in such without clinical, endoscopic, biochemical and histological disease activity. The sensitivity of the pipelines allows detection of subtle morphologic alterations that warrant further investigation, as does the usage of G3BP1 as an inflammatory bowel disease stress marker. *(Cell Mol Gastroenterol Hepatol 2026;20:101680; https://doi.org/10.1016/j.jcmgh.2025.101680)*

Keywords: Automated Image Analysis; CellProfiler; G3BP1; Inflammatory Bowel Disease; Quantitative Analysis; Stress Granules.

Intestinal epithelial cells (IECs) form the inner lining of the gastrointestinal wall and play a pivotal role in maintaining gut homeostasis by facilitating numerous functions, such as nutrient absorption, electrolyte balance, endocrine activity, link to the nervous system, and mucosal immunity. IECs are essential for orchestrating interactions with immune cells residing in the gut-associated lymphoid tissue.[1,2] The intricate crosstalk between IECs and immune cells is essential for controlling inflammation and protecting cells against pathogens and other invaders.[3] Conditions such as Crohn's disease (CD) and ulcerative colitis (UC), also known as inflammatory bowel diseases (IBDs), are characterized by chronic inflammation of the intestinal wall due to an altered immune reaction.[4] Investigating and understanding the interactions between IECs and immune cells is vital for unraveling the mechanisms that underlie such diseases and for the development of effective treatments. Laser-scanning confocal imaging is a powerful tool for studying the intestinal epithelium and immune cell distribution, contributing to quantitative biological data. However, assessment and accurate segmentation of cells and the measurement of cellular features in whole tissue sections can be challenging due to complex heterogeneity of the tissue and the variation in cell morphology. Recently, several studies have highlighted the versatility of CellProfiler in segmenting different cell types and cellular features for further downstream analyses.[5–7] Designing and publishing automated pipelines offers numerous advantages in the field of cell biology. These pipelines not only facilitate the analysis of a large number of images in a short period of time but also allow the investigation of multiple regions within a single organ or tissue. To date, only one study has addressed the challenge of deciphering distinct cell types within complex gastrointestinal tissues using a combination of different software tools.[8] More precisely, the methodology incorporated semantic machine learning to map mononuclear phagocyte-T cell interactions within mouse Peyer's patches, as well as to identify and quantify distinct populations of intraepithelial lymphocytes in rat jejunum.

In this study, we developed a novel and user-friendly approach to analyze immunofluorescence staining of intestinal tissue sections using the free software tool CellProfiler.[9] Herein, we designed 2 automated pipelines capable of: (1) distinguishing between distinct IEC types from cells within the lamina propria; and (2) facilitating intracellular protein quantification. These pipelines are free to use and can be adapted to different organ structures and proteins of interest. Notably, no specific programming skills are required to utilize these pipelines. The EpiCellProfiler (ECP) pipeline was designed to mark IECs within intestinal tissue sections, enabling separation of the mucosa from lamina propria cells. This pipeline is particularly useful for downstream analysis, focusing on crypt structures under inflamed or non-inflamed conditions. The PropiCellProfiler (PCP) pipeline indirectly identifies lamina propria cells by excluding IECs marked with the epithelial cell adhesion molecule (EpCAM). In addition, the PCP pipeline includes several nuclear morphological parameters, as changes in nuclear shape are associated with activity states[10] and cellular senescence.[11,12] Moreover, both pipelines are designed to evaluate the intensity, distribution, and localization of Ras GTPase-activating protein-binding protein 1 (G3BP1), a multifunctional protein within the stress granule cycle and a molecular stress marker.

G3BP1 is a multifaceted protein involved in diverse biological functions.[13,14] It is predominantly found in the cytoplasm, where it acts as a molecular switch, triggering RNA-dependent phase separation to assemble stress granules (SGs).[15] Recently, it has been demonstrated that overexpression alone can dominantly induce SG formation even in the absence of stress.[16] Moreover, the presence of the nuclear transport factor 2 motif (NTF2 domain) at the C-terminus associates G3BP1 with nuclear transport.[17,18] Although direct evidence for the dynamic translocation of G3BP1 under certain conditions is limited, its interaction with proteins, such as p53, suggests additional potential nuclear involvement.[19,20] SGs have been shown to contribute to the pathogenesis of several human diseases; however, their role in gastrointestinal tissues and their association with IBD remains unknown. The segmentation of different cellular compartments and the ability to extract data from individual cells based on various parameters make CellProfiler useful for evaluating G3BP1 levels in the cytoplasm and nucleus and elucidating the localization of cells in the intestinal mucosa.

In summary, we described customized, fully automated CellProfiler-based pipelines for high-throughput quantitative analysis of images acquired by laser-scanning confocal microscopy of fluorescently labeled histological colonic tissue samples. Our pipelines allow rapid and accurate analysis of thousands of cells across multiple gastrointestinal regions and the generation of multiple cellular morphology parameters at the single-cell level. In addition, they register the localization and distribution of biological markers associated with cellular stress. Thus, we provide a user-friendly

Abbreviations used in this paper: **3D, 3-dimensional; ANOVA, analysis of variance; CD, Crohn's disease; cIBD, colonic inflammatory bowel disease; CRP, C-reactive protein; DAPI, 4′,6-diamidino-2-phenylindole; ECP, EpiCellProfiler; EpCAM, epithelial cell adhesion molecule; G3BP1, Ras GTPase-activating protein-binding protein 1; IBD, inflammatory bowel diseases; IECs, intestinal epithelial cells; IFN, interferon; mRNP, messenger ribonucleoprotein; NTF2, nuclear transport factor 2; P/S, penicillin-streptomycin; PBs, processing bodies; PBST, phosphate buffered saline with Tween 20; PCP, PropiCellProfiler; RNP, ribonucleoprotein; RT, room temperature; SEM, standard error; SES-CD, Simple Endoscopic Score for Crohn's Disease; SGs, stress granules; STED, stimulation emission depletion; TNF, tumor necrosis factor; UC, ulcerative colitis.**

Most current article

© 2025 The Authors. Published by Elsevier Inc. on behalf of American Gastroenterological Association Institute. This is an open access article under the CC BY license (http://creativecommons.org/licenses/by/4.0/).
2352-345X
https://doi.org/10.1016/j.jcmgh.2025.101680

platform that allows for the exploration of spatial and subcellular distribution of markers relevant to intestinal health and disease, particularly in the context of IBD.

Results

ECP Revealed Distinct G3BP1 Abundance Patterns in Control Tissues in Patients With CD and UC in Remission

Our ECP pipeline effectively segmented IECs within crypts and enabled the quantification of G3BP1 distribution and abundance across different cellular compartments. Immunofluorescence panels show representative images of colonic tissue section with G3BP1 distribution from control, CD, and UC in remission samples (Figure 1*A*). Quantitative analysis revealed significantly higher integrated G3BP1 pixel intensities (Figure 1*B*) and average G3BP1 pixel intensities (Figure 1*C*) in patients with CD and UC in remission compared with controls. Notably, patients with UC in remission displayed significantly elevated G3BP1 levels in both measures when compared with controls (both $P <$ .0001). In contrast, no substantial differences emerged between UC and CD in remission (B: P =.0142; C: $P =$ .2325). Visual inspection of the pseudo-colored intensity map demonstrated a uniform distribution of the G3BP1 signal throughout epithelial cells, particularly in close proximity to the cell nucleus, across all groups. Importantly, a marked increase in the frequency of G3BP1 granules was observed in patients with CD and UC in remission (Figure 1*D*). Subcellular analysis revealed that nuclear and cytoplasmic G3BP1 intensities were significantly higher in patients with CD and UC compared with controls, with the most pronounced increase observed in UC in remission (Figure 1*E*). Within the control group, nuclear and cytoplasmic G3BP1 levels displayed no significant difference, whereas in both UC and CD in remission, G3BP1 levels in the cytoplasm significantly exceeded nuclear levels (CD: $P =$ .0006; UC: $P =$ .0051). G3BP1 intensity was particularly increased in the nuclei of UC in remission samples when compared with control nuclei ($P =$ .006), whereas this increase was absent in CD in remission nuclei. Cytoplasmic G3BP1 levels, however, were significantly elevated in both UC ($P <$ .0001) and CD ($P =$ .0005) in remission when compared with controls. To complement this analysis, we assessed the number of G3BP1 spots across groups and compartments (Figure 1*F*). Both UC and CD in remission displayed significantly increased G3BP1 spot counts compared with controls, despite clinical and histological remission ($P <$ .0001). When comparing nuclear compartments, UC in remission displayed significantly higher nuclear spot counts compared with controls ($P =$ .0013), whereas CD in remission samples showed no significant difference. Given that CellProfiler assigns a 'child' object to a 'parent' object based on maximum overlap, spots at the nuclear edge may be classified as nuclear rather than cytoplasmic. To address this, we quantified G3BP1 intensity exclusively in the cytoplasm across 3 radial bins to analyze its spatial distribution (Figure 1*G*). Although total G3BP1 intensity was elevated in CD and UC samples, the fractional intensity distribution remained largely unchanged between CD and UC and control groups. In all groups, the highest fraction of G3BP1 intensity was found in the peripheral cytoplasm (bin 3), a finding partially attributable to the larger area of this compartment relative to other bins (Figure 1*H*). Further analysis of G3BP1 spot size revealed significantly larger granules in CD ($P =$ .0029) and UC ($P <$ .0001) in remission compared with controls (Figure 1*I*).

Super Resolution Confirms Subcellular Localization of G3BP1 in Colonic Epithelium

High-resolution stimulation emission depletion (STED) imaging corroborated these findings, demonstrating that the increased G3BP1 intensity was not linked to preferential peripheral localization but rather reflected a homogeneous distribution throughout the cytoplasm with more prominent and aggregated spots in CD and UC samples (Figure 2*A*–*C*). This suggests that the observed increase in G3BP1 intensity is driven by a global upregulation and accumulation of the protein. Finally, a 3-dimensional (3D) video reconstruction of IECs processed with the Huygens imaging platform provided a comprehensive volumes view of G3BP1 granule distribution within the cell, emphasizing their increased abundance and molecular condensation in samples from patients with CD and UC around the nucleus. These high-resolution analyses provide orthogonal confirmation of our quantitative pipeline-based findings and strengthen the evidence that epithelial cells in IBD are subjected to abnormal SG dynamics.

Automated Analysis Using the PCP Pipeline Reveals Altered Nuclear Morphology and Increased G3BP1 Expression in Patients With UC in Remission

To investigate G3BP1 dynamics in post-inflammatory colonic tissue, we employed CellProfiler to quantify G3BP1 spots and examine their size and abundance in cells relevant to gut homeostasis and immune function (Figure 3*A*). Using the PCP pipeline, analysis of G3BP1-positive cells revealed a significantly higher proportion in UC in remission (84.40%) compared with controls (71.93%; $P =$ .0055), whereas no significant difference was observed between UC and CD in remission (81.51%; ns) (Figure 3*B*). A similar pattern emerged when analyzing G3BP1 spot counts per cell in the lamina propria. UC in remission exhibited the highest number of G3BP1 spots per cell (median, 9.82), significantly exceeding both CD in remission (median, 6.34; $P =$ .0182) and controls (median, 6.15; $P =$ .0003) (Figure 3*C*). Additionally, UC in remission showed significantly elevated integrated G3BP1 intensity (Figure 3*D*) and mean intensity per cell (Figure 3*E*) compared with controls (both $P <$ .0001) and CD in remission (D: $P =$ .0035; E: $P =$ .0198). Although CD in remission displayed less pronounced differences relative to controls, significant increases in G3BP1 intensity were still detected (D: $P =$ .0414; E: $P =$ .0182). G3BP1 spot size analysis further demonstrated enlarged granules in both UC (median, 16.50) and CD (median, 16.84) in remission

compared with controls (median, 15.27; $P = .0003$ and $P = .0101$) (Figure 3*F*). These findings collectively suggest altered SG dynamics and G3BP1 persistence in patients with CD and UC despite clinical remission. During confocal imaging for G3BP1 quantification, we observed distinct nuclear morphological differences in UC samples compared with controls. Alterations in nuclear shape, size, and compactness are widely recognized as hallmarks of cellular stress, activation, or apoptosis. To systematically analyze nuclear morphology, we integrated the *MeasureObjectSizeShape* module into the PCP pipeline, enabling precise assessment of nuclear dimensions in immune-relevant cells. UC in remission samples exhibited significantly larger nuclear areas compared with both controls and CD in remission (both $P < .0001$) (Figure 3*G*). No significant differences were observed between CD in remission and

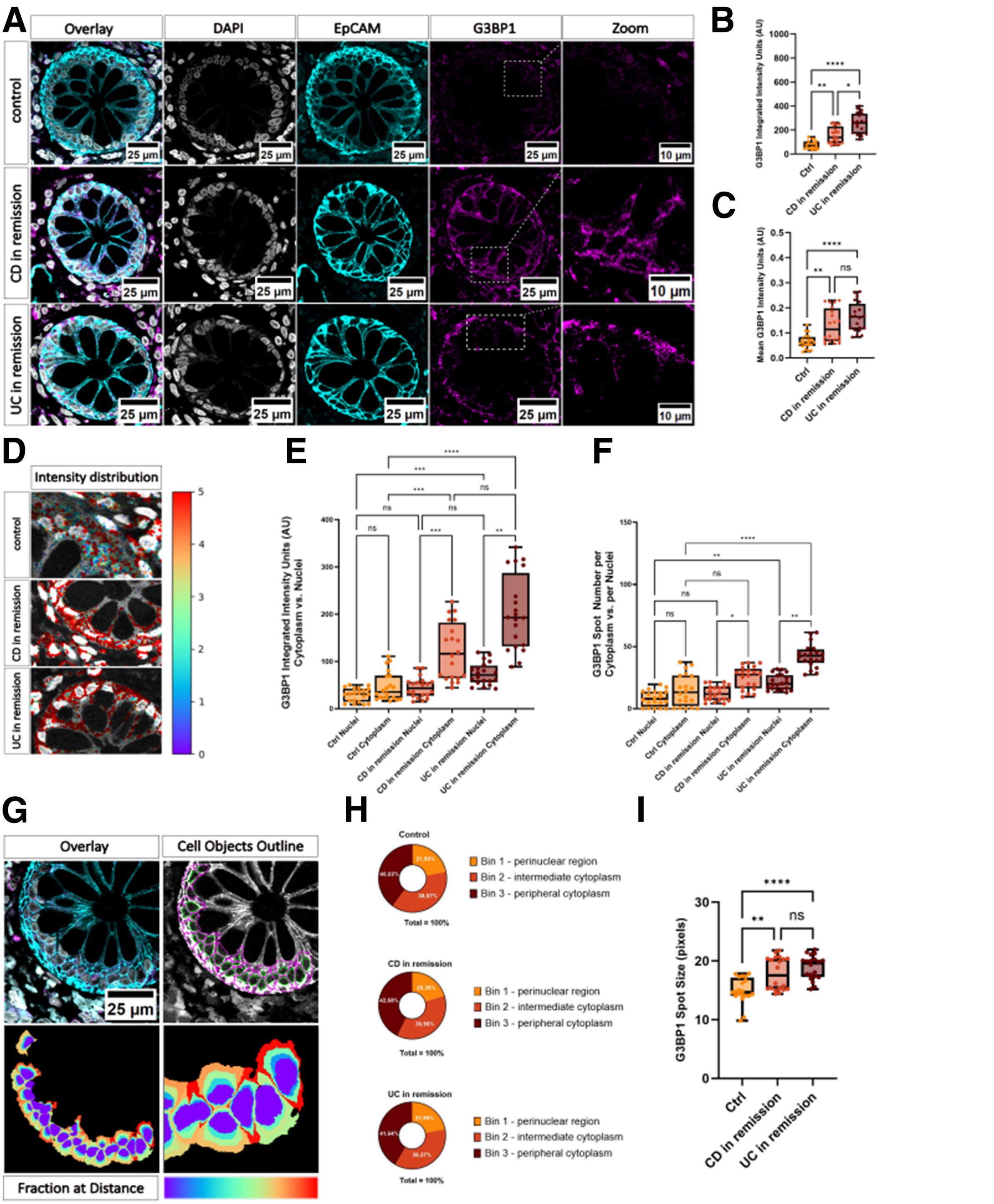

controls (ns). Nuclear circularity analysis revealed more irregularly shaped nuclei in UC in remission compared with both controls ($P = .0087$) and CD in remission ($P = .0177$) (Figure 3*H*). Consistent with this, mean nuclear perimeter (Figure 3*I*) and mean nuclear radius (Figure 3*J*) were significantly increased in patients with UC compared with both controls and CD in remission (both $P < .0001$). Nuclear compactness, reflecting less smooth and more irregular nuclear contours, was significantly reduced in UC in remission compared with both controls ($P = .0110$) and CD in remission ($P = .0320$) (Figure 3*K*).

Collectively, these findings reveal substantial alterations in nuclear morphology and increased G3BP1 expression in patients with UC in remission, suggesting persistent cellular stress or structural reorganization in the post-inflammatory colonic microenvironment. The successful implementation of the PropiCellPipeline emphasizes its versatility and effectiveness for investigating complex cellular structures, making it a valuable tool for future research in inflammatory conditions.

Validation and Comparison of Automated Cell Counting by CellProfiler vs Manual Analysis and ImageJ Software

Both pipelines were developed to address the need for automated cell counting in complex gastrointestinal tissues. Both pipelines were designed to overcome segmentation challenges that arise in densely packed regions, ensuring robust and accurate quantification. By integrating advanced filtering mechanisms, our pipelines improve data precision by minimizing the impact of over-segmentation errors, which are common in traditional image analysis methods. To assess the accuracy and reliability of our pipelines, we compared CellProfiler results with manual counting. Regression analysis demonstrated that the PCP pipeline achieved a stronger correlation with manual counting ($R^2 = 0.8612$) than ECP ($R^2 = 0.7476$), underscoring the precision performance of PCP in complex tissue environments (Figure 4*A* and *B*). Bland-Altman analysis for both pipelines indicated a negative mean bias (PCP, −10.11; ECP, −9.94), suggesting a consistent underestimation of cell counts relative to manual counting (Figure 4*C* and *D*). This underestimation is likely attributed to the filtration of incorrectly segmented cells within the workflow, ensuring that erroneous objects are excluded from the final analysis to improve data integrity (Figure 4*E*). To compare G3BP1-positive signals, we utilized the *IdentifyPrimaryObject* module in CellProfiler and the Analyze Particle module in ImageJ on identical image datasets (Figure 4*F*). A total of 136 images were analyzed using comparable settings and thresholds. Bland-Altman analysis demonstrated stronger agreement for images containing fewer than 5000 G3BP1 spots per image (mean bias, −2.329). For images with between 5000 and 10,000 spots, discrepancies increased slightly (mean bias, −3.204), whereas images exceeding 10,000 G3BP1 spots showed greater variability and instances of over- or underestimation by CellProfiler (mean bias, 4.148) (Figure 4*G*). These findings highlight that those discrepancies become more pronounced with increasing spot counts, reinforcing the importance of understanding software-specific limitations in dense tissue environments. Despite these differences, our results confirm that both PCP and ECP provide robust and reproducible cell counting data. Importantly, the improved accuracy of PCP compared with ECP emphasizes its suitability for investigating cellular features in complex gastrointestinal tissues. By addressing common segmentation challenges and enhancing filtering steps, the PCP pipeline offers a precise and efficient tool for quantitative cellular analysis. Future studies may further refine these pipelines to expand their application in diverse tissue types and disease contexts.

Staining Variability Influence the Robustness of the Image Analysis Pipeline

To evaluate the impact on the performance of our image analysis pipelines in terms of segmentation, we first

Figure 1. (***See previous page***). **Representative images and analyses of the expression of G3BP1 within intestinal crypts of human colon tissue sections.** (*A*) Laser-scanning confocal micrographs of colonic epithelial tissue from a control, a patient with CD, and a patient with UC in remission. Sections were stained for the epithelial marker EpCAM (*cyan*), the nuclear marker DAPI (*gray*), and G3BP1 (*magenta*). The *left panels* show merged images, followed by individual channels for EpCAM, DAPI, and G3BP1. Scale bar: 25 μm. *Right panels* are higher magnification views, highlighting the subcellular distribution of G3BP1, indicated by the *dashed boxes*. (*B*) Integrated and (*C*) mean G3BP1 fluorescence intensity (arbitrary units, AU) per crypt reveals significant decrease in total G3BP1 signal in control tissue compared with CD and UC in remission. (*D*) Representative confocal images illustrating the spatial intensity distribution of G3BP1 (pseudo colored from low [*blue*] to high [*red*] intensity) in colonic crypt cross-sections. Nuclear regions appear in *gray*, whereas G3BP1 signal intensity is overlaid using the color scale bar. (*E*) Integrated G3BP1 fluorescence intensity (arbitrary units, AU) shows significant differences in the nuclear vs cytoplasmic compartments in CD and UC cells compared with control. (*F*) Number of G3BP1 spots per nucleus vs per cytoplasm for each group, showing that tissues from patients with CD and UC in remission exhibit altered G3BP1 formation compared with controls. (*G*) *Left panel* shows merged images, whereas the *right panel* outlines the identification of nuclei (*green*) and identification of cell edges (*magenta*) made by CellProfiler. Scale bar: 25 μm. The lower panel illustrates a 'fraction-at-distance' (FracAtD) heat map, subdividing the cytoplasm into 3 radial bins (bin 1 = perinuclear, bin 2 = intermediate, bin 3 = peripheral cytoplasm) based on increasing distance from each nucleus object. (*H*) Stacked bar charts quantifying the relative fraction (%) of total G3BP1 intensity within each radial bin for control and patients with CD and UC in remission. (*I*) G3BP1 spot size (in pixels) for each group showing larger G3BP1 spots in patients with CD and UC in remission, compared with control cells. Data represent mean values calculated from 20 images per group (Data are based on N = 5 patients per group, with n = 4 images analyzed per patient). Statistical significance was assessed using a nonparametric Kruskal-Wallis test. Significance levels were indicated as follows: ns = not significant; *$P < .05$; **$P < .01$; ***$P < .001$; and ****$P < .0001$.

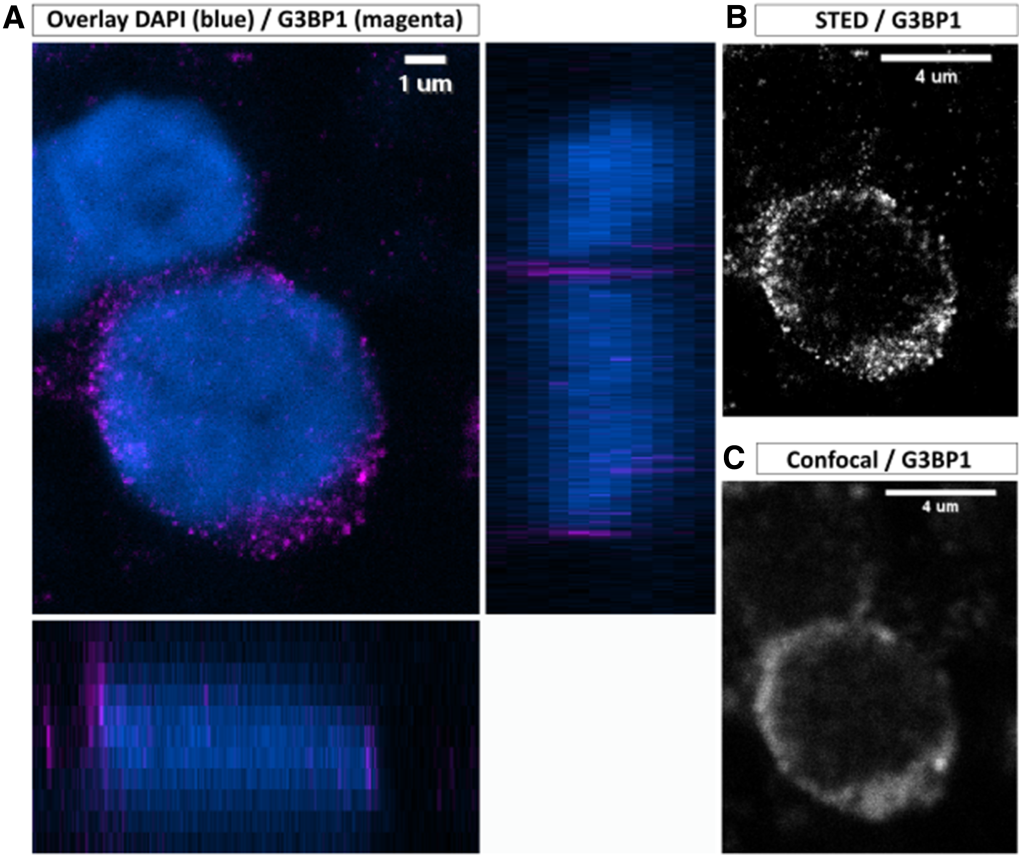

Figure 2. STED microscopy revealed localization of G3BP1. (*A*) Immunofluorescent detection of G3BP1 (STAR RED) and DAPI (*blue*) were visualized in unaffected UC colonic specimen, imaged by both confocal (DAPI) and STED (G3BP1) microscopy. Image at the bottom showing Z-projection in the X–Z direction and on the right side in the Y–Z direction. *White lines* indicate the Z-depth of the slice. Z-stack images were collected every 0.33 μm. Total size, 4 μm. Scale bar is 1 μm. (*B* and *C*) Comparison between STED and confocal visualization of G3BP1 granules, showing an increased molecular condensation around the nucleus. Scale bar is 4 μm.

selected representative images of intestinal tissue sections that had been immunostained for EpCAM (Figure 5*A*). To objectively categorize images as having either high or low staining quality, we extracted pixel intensity distributions from selected images and overlaid the resulting histograms (Figure 5*B*). High-quality images displayed a sharp and distinct intensity peak corresponding to epithelial regions, whereas low-quality images showed broader and less defined intensity ranges. These characteristics of the histograms served as the basis for categorizing the images into groups of high and low staining quality for further analysis.

To systematically evaluate the image features that predict successful segmentation, we employed CellProfiler's *MeasureImageQuality* module to correlate various image metrics with segmentation deviation. Segmentation deviation was defined as the absolute difference in cell counts between manual annotation and automated segmentation. Pearson correlation analysis revealed that the deviation between automated and manual segmentation significantly correlates with EpCAM-specific quality metrics, particularly the focus score (r = −0.66; R^2 = 0.44; P = .0135) and standard intensity (r = −0.56; R^2 = 0.31; P = .0462), indicating that both image sharpness and signal variability are critical factors for accurate segmentation. In contrast, 4′,6-diamidino-2-phenylindole (DAPI)-related metrics showed only weak or no correlation with segmentation performance (Figure 5*C*).

Next, we compared the segmentation deviation in images of varying staining quality (Figure 5*D*). Low-quality images exhibited significantly higher segmentation deviation, with an average error of 10.38%, compared with 2.47% for high-quality images. A grouped analysis using a boxplot representation (Figure 5*E*) further supported this finding, confirming a statistically relevant increase in segmentation error for low-quality images ($P < .01$).

From Skin to Stomach: Evaluating the Versatility of the EPC and PCP Pipelines Across Multiple Human Tissues

To evaluate the versatility of our CellProfiler-based segmentation workflow beyond colonic samples, we ran the pipeline on various human tissues without adjusting its settings. Robust epithelial segmentation was achieved in mammary gland, stomach, kidney, and skin samples

(Figure 6). In these tissues, the presence of well-defined epithelial compartments expressing EpCAM enabled the accurate identification of epithelial regions from their surrounding stromal environment. Expression of EpCAM in the adult human kidney was restricted to tubular compartments. Glomeruli were generally EpCAM-negative; however, the overall segmentation performance was limited within the kidney due to the heterogenous background signal from non-epithelial structures such as blood vessels. By contrast, the liver lacks clearly defined epithelial structures and exhibits a more diffuse arrangement of single epithelial and stromal elements. Overall, these findings demonstrate the versatility of CellProfiler as a segmentation platform, while emphasizing that its performance depends heavily on the structural organization of the target tissue and the appropriateness of the chosen

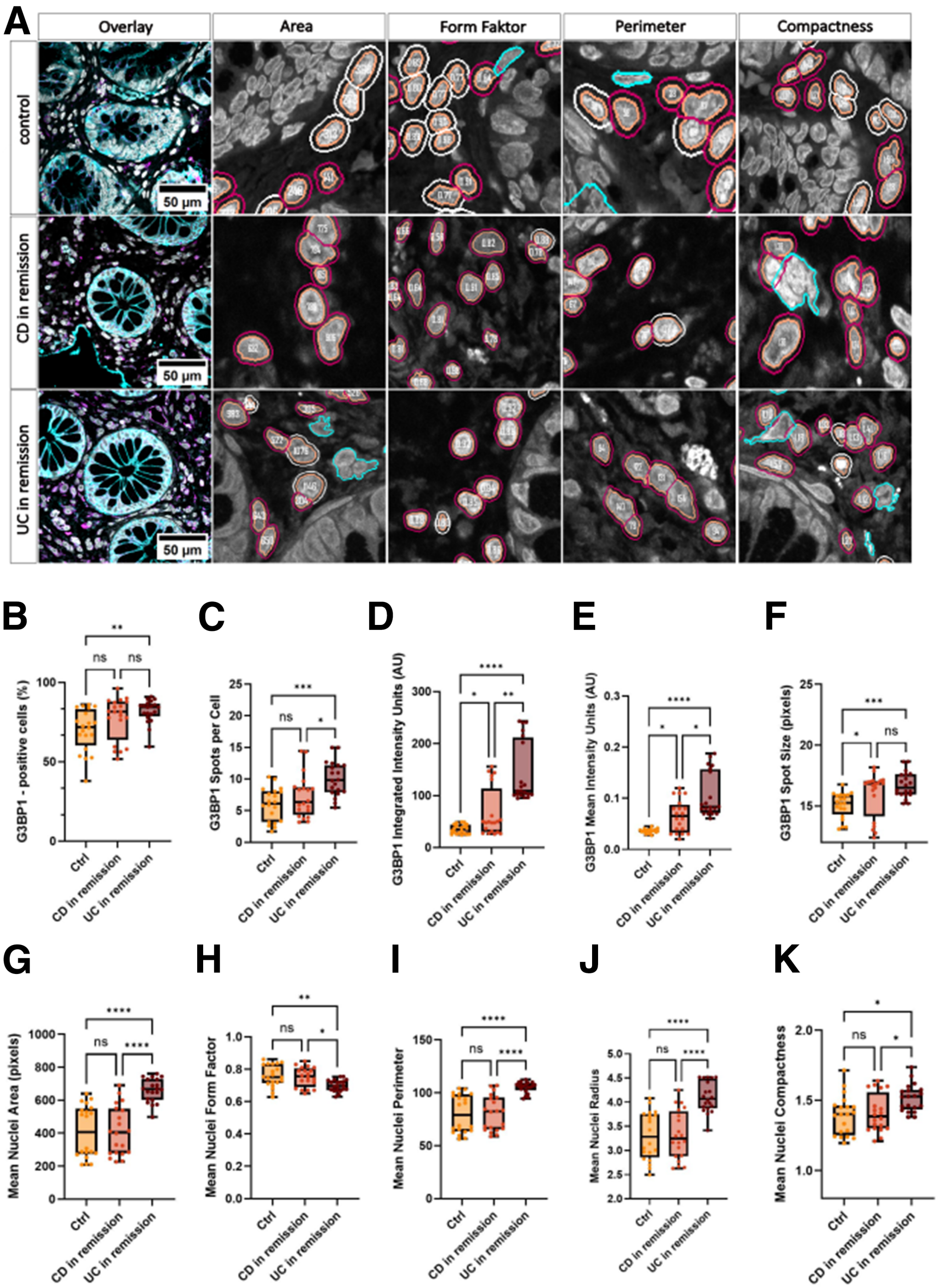

segmentation marker for the biological question under investigation.

Discussion

Herein, we demonstrate the usability and efficacy of the open-source software tool CellProfiler in automatically segmenting intestinal tissue and cell types from confocal microscopy images captures. This capability facilitates the independent assessment of diverse quantitative and qualitative measurements on tissue, cell, and subcellular levels. The human intestine is a complex immunological environment and contains the largest number of immune cells of any tissue in the body. The function of the intestinal immune system in the steady-state is to maintain homeostasis despite constant environmental stressors. Separated by a thin basement membrane, the lamina propria and epithelium represent very distinct compartments harboring specific immunological processes.[21] Besides the segmentation of IECs and cells within the lamina propria, our study utilized the RNA stress protein G3BP1 to compare levels of molecular stress between patients with CD and UC in clinical, endoscopic, and histological remission with non-IBD controls. G3BP1 is a multifaceted protein and a characteristic component in the formation of membrane-less messenger ribonucleoprotein (mRNP) particles known as SGs.[22,23] The aberrant assembly or disassembly of these intracellular structures has been shown to be implicated in pathological conditions such as neurodegenerative diseases,[24] cancer,[25] age-related conditions,[26] and viral infections.[27,28] However, little is known about the role of SGs in chronic stress conditions such as IBD. Initially, the role of SGs in modulating inflammatory responses by controlling the stability and translation of key cytokine mRNAs has been explored in several studies. For instance, research has shown that pro-inflammatory cytokines like interferon (IFN)-γ and tumor necrosis factor (TNF)-α can enhance SG formation in IECs, leading to the sequestration and translational inhibition of specific mRNAs, such as Hsp70.[29] Proteomic and transcriptomic analyses of SGs and processing bodies (PBs) in human T lymphocytes have further revealed that these granules dynamically reorganize upon immune activation.[30] These findings suggest that SGs play a significant role in the post-transcriptional regulation of cytokine expression, thereby modulating inflammatory responses in conditions like IBD. Furthermore, the intracellular distribution of certain proteins can have a significant impact on cellular phenotypes and their functional consequences. For example, LC3, a marker for autophagosomes, shows a homogeneous pattern under basal conditions, whereas local accumulation often indicates impaired autophagy.[31,32] This shows that changes in protein distribution, rather than abundance alone, can reflect altered cellular states. In line with this, the aggregation of G3BP1 granules observed in our study may reflect a dysregulated stress response rather than a mere increase in overall G3BP1 abundance. This highlights the importance of analyzing both the abundance and spatial distribution of key molecular markers to better understand cellular stress mechanisms.

Our findings extend this concept by revealing distinct patterns of G3BP1 distribution in tissues from patients with CD and UC. We observed not only a general upregulation and homogeneous accumulation of G3BP1, but also significant differences in its cellular localization between patient groups. The aggregation of G3BP1 spots, demonstrated by both quantitative image analysis and confirmatory high-resolution STED microscopy, may indicate an abnormal stress response and compromised epithelial barrier function, promoting chronic inflammation as a hallmark of IBD. Notably, the persistence of G3BP1 granules in patients classified as being in histological remission may point toward residual inflammation at the molecular level, suggesting that IECs remain under stress even when overt inflammatory infiltrates are absent. This aligns with previous observations that residual endoscopic inflammation can persist despite clinical remission and can be predicted by laboratory indicators such as elevated C-reactive protein (CRP), white blood cell count, and short remission duration.[33] In vitro studies will be necessary to further define the functional role of G3BP1 granules in IECs and to clarify whether they act as passive markers of stress or actively contribute to disease processes.

Previously, studies have highlighted the critical role of G3BP1 in the development and progression of various

Figure 3. (***See previous page***). **Laser-scanning confocal micrographs and CellProfiler output measurements of Mean Nuclei Area, Form Factor, Perimeter, Radius and Compactness of lamina propria cells from control, patients with CD and patients with UC in remission.** (*A*) Sections were stained for the epithelial marker EpCAM (*cyan*), the nuclear marker DAPI (*gray*), and G3BP1 (*magenta*). The *left panels* show merged images, followed by individual output measurements of CellProfiler. Cells in cyan were excluded from the measurements for 3 possible reasons: Their cell size is too large, which possibly indicates incorrect segmentation, cells overlap with the EpCAM staining and are therefore considered as IECs, or a cell is touching the border of the image. Scale bar: 50 μm. (*B*) Cells were imaged, masked, segmented, and the percentage of cells positive for G3BP1 was calculated as (cell count G3BP1-positive) / (total cell count) $\times$ (100). (*C*) Average G3BP1 spots per cell objects. (*D*) Sum of total G3BP1 pixel intensities within cell objects. (*E*) Average G3BP1 pixel intensity within cell objects. (*F*) Average area (in pixels) of identified G3BP1 spots within cell objects. (*G*) Mean nucleus area, measured as the number of pixels in delineated primary nuclei objects. (*H*) Mean form factor calculated as ($4 \times \pi \times$ Area/Perimeter^2). (*I*) Mean nucleus perimeter, calculated as the total number of pixels in distinct areas within the image. (*J*) Mean nuclei radius, measured as the mean distance of any pixel in the object to the closest pixel outside of the object. (*K*) Mean nuclei compactness. Value of 1 describes how closely a shape resembles a perfect circle. Data represent mean values calculated from 20 images per group (Data are based on N = 5 patients per group, with n = 4 images analyzed per patient). Statistical significance was assessed using a nonparametric Kruskal-Wallis test. Significance levels were indicated as follows: ns = not significant; **P* $<$ .05; ***P* $<$ .01; ****P* $<$ .001; and *****P* $<$ .0001.

cancers, including cancers of the human digestive system.[34] G3BP1 expression has been found to be significantly upregulated in colon cancer tissues and cells compared with controls.[35] Additionally, the study showed that elevated G3BP1 levels are strongly linked to a poor prognosis and more advanced stages of colon cancer in these patients. Elevated G3BP1 expression levels have been associated with similar poor outcomes in other cancers, including gastric cancer[36,37] and non-small-cell lung cancer.[38] Patients with longstanding colonic IBD (cIBD) have an increased risk of developing colorectal dysplasia or even colitis-associated cancer compared with the general population.[39,40] Together with the results of our study, showing the high expression of G3BP1 in patients with UC and CD in remission, G3BP1 may serve as a potential biomarker to assess the risk of disease progression from chronic

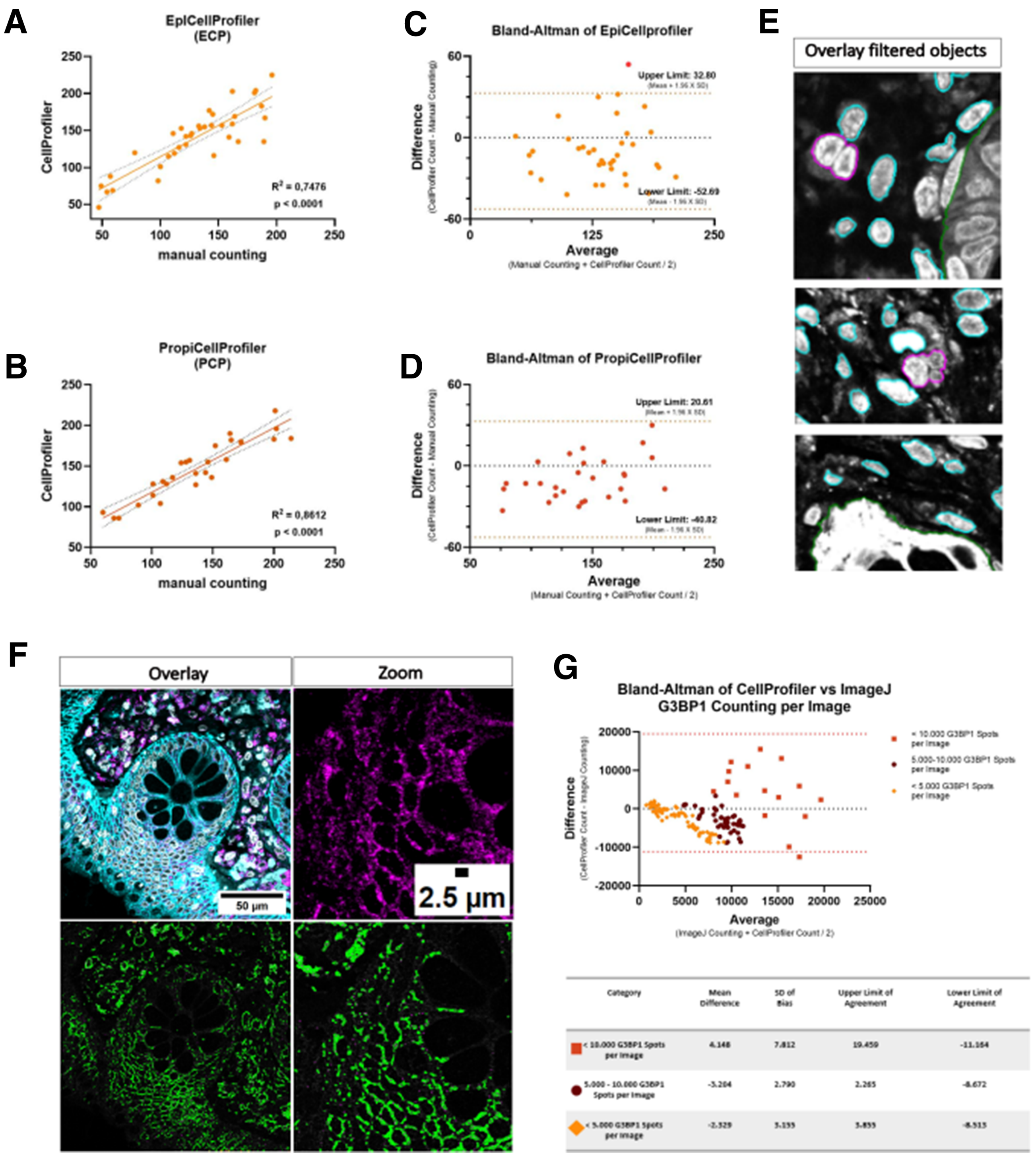

Category	Mean Difference	SD of Bias	Upper Limit of Agreement	Lower Limit of Agreement
< 10.000 G3BP1 Spots per image	4.148	7.812	19.459	-11.164
5.000 - 10.000 G3BP1 Spots per image	-3.204	2.790	2.265	-8.672
< 5.000 G3BP1 Spots per image	-2.329	3.155	3.855	-8.513

inflammation to neoplasia. Monitoring G3BP1 levels in patients with UC and CD during remission could help identify those at higher risk for both relapsing inflammatory periods and potential malignant transformation, but further research is needed to validate its reliability as a potential biomarker.

Alterations in the nuclear morphology have long been recognized as hallmarks of cellular transformation and disease progression, particularly in cancer, where nuclear envelope remodeling is associated with genomic instability and altered gene regulation.[41–43] Using the *MeasureObjectSizeShape* module on nuclear objects within our PCP pipeline, we quantitatively extracted morphometric features to describe the shape and size of cell nuclei. A notable observation was the presence of nuclear enlargement and irregular nuclear shapes of immune cells in rectal biopsies of UC patients in remission which was not mirrored in CD, yet following the natural phenotype of this disease. The mechanistic drivers of nuclear abnormalities and the potential functional consequences are incompletely understood. Noteworthy, disruption of nuclear envelope integrity has been shown as initiating event in tauopathies and were linked to nuclear maintenance and repair mechanisms during tau aggregation.[44] Given the well-established association between SGs and neurodegenerative diseases, it remains unclear whether dysregulated SG formation is a consequence of aberrant nuclear morphology or whether nuclear abnormalities arise from impaired SG dynamics. In UC, abnormal nuclear morphology may mark impaired epithelial and immune cell function. Studies have shown that epithelial regeneration remains compromised in UC, even after inflammation resolves, suggesting a persistent defect in tissue repair mechanisms.[45] Moreover, epithelial regeneration is critical for barrier integrity and nutrient absorption, and reduced regenerative capacity is considered a hallmark of intestinal ageing, a process that may be accelerated in UC due to repeated inflammation and mucosal damage.[46] Cellular senescence, which is characterized by cell cycle arrest, nuclear enlargement, and altered chromatin organization, emerges as a plausible mechanistic link. Senescent epithelial or immune cells can adopt a proinflammatory secretory phenotype, known to sustain low-grade inflammation and impede tissue regeneration.[47] Thus, the nuclear dysmorphisms observed in UC may reflect a combination of cellular senescence, DNA damage, and regenerative failure, factors that contribute to persistent mucosal dysfunction and chronic disease progression, even in remission. Although multiple studies have suggested diverse functions of G3BP1, ranging from SG nucleation to the regulation of RNA metabolism to the senescence-associated secretory phenotype,[48] its exact role in epithelial cells within the context of CD and UC remains unclear. Dedicated mechanistic studies will therefore be required to disentangle whether G3BP1 primarily acts as a marker of cellular stress, a driver of proinflammatory signaling, or both.

Segmenting nuclei is highly useful for a number of biological tasks, including the quantitative analysis of cellular composition in any tissue. However, it poses significant challenges due to cell shape variability and the presence of overlapping or closely packed regions. The accuracy of the segmentation is critical for the reliability of downstream analyses. In cases where nuclear staining is suboptimal, segmentation quality may be compromised, potentially leading to incorrect data interpretation. For example, oversegmented cells do not accurately represent the morphology of individual cells and cannot be fully excluded within an automated analysis, particularly in complex tissue section. As demonstrated in previous studies, an error rate of up to 10% per image can be expected. We therefore focused on the contribution of staining variability to the segmentation performance of our pipelines. Our results show that the accuracy strongly depends on the choice and quality of the marker staining. Images with low EpCAM staining intensity showed significantly greater deviation between manual and automated counts (10.38%) than high-quality images (2.47%). These results emphasise the importance of consistent and robust staining for achieving reliable segmentation. We recommend that researchers carefully optimize and standardize their staining protocols prior to applying automated segmentation workflows. However, to address the issue of staining variability and to further increase the precision of our data analysis, we have implemented 2 key strategies in

Figure 4. (***See previous page***). **Linear regression analysis comparing CellProfiler-based quantification with manual counting or ImageJ software.** Pearson's correlation and simple linear regression were performed on 36 paired image measurements for (*A*) lamina propria cells (coefficient = 0.8612; n = 28 images analyzed) and (*B*) IECs (coefficient = 0.7476; n = 36 images analyzed), to assess the comparability of methods. (*C and D*) display scatter diagrams of the difference plotted against the averages of 2 measurements (*C*: mean bias of −10.11 and *D*: mean bias of −9.94). *Horizontal lines* are drawn at the mean difference and at the limits of agreement. Each *orange dot* represents a paired measurement difference between the 2 methods, plotted against the average. *Red dots* represent outliers. Most data points fall near the zero line, indicating overall agreement between manual counting and cell counting with CellProfiler. (*E*) Graphical representation of cells that were filtered and removed in the pipeline. Cells in *magenta* were excluded from the measurements for 3 possible reasons: Their cell size is too large, which possibly indicate incorrect segmentation, cells overlap with the EpCAM staining and are therefore considered as IECs, or a cell is touching the border of the image. (*F*) Output images after analysis in CellProfiler and ImageJ showing all G3BP1-counted objects per image. Scale bars represents 25 μm and 2.5 μm. (*G*) Plot of differences between CellProfiler and ImageJ G3BP1 spot count vs the mean of the 2 measurements. The scatter plot illustrates that the discrepancy between the G3BP1 spot count in CellProfiler and ImageJ is minimal when the spot count per image is low (mean bias of −2.329), and it increases as the spot count per image rises (mean bias of 4148). The bias and limits of agreement were calculated with 96% confidence intervals (CIs), and results are summarized in the table below. Per group, 74 images were analyzed.

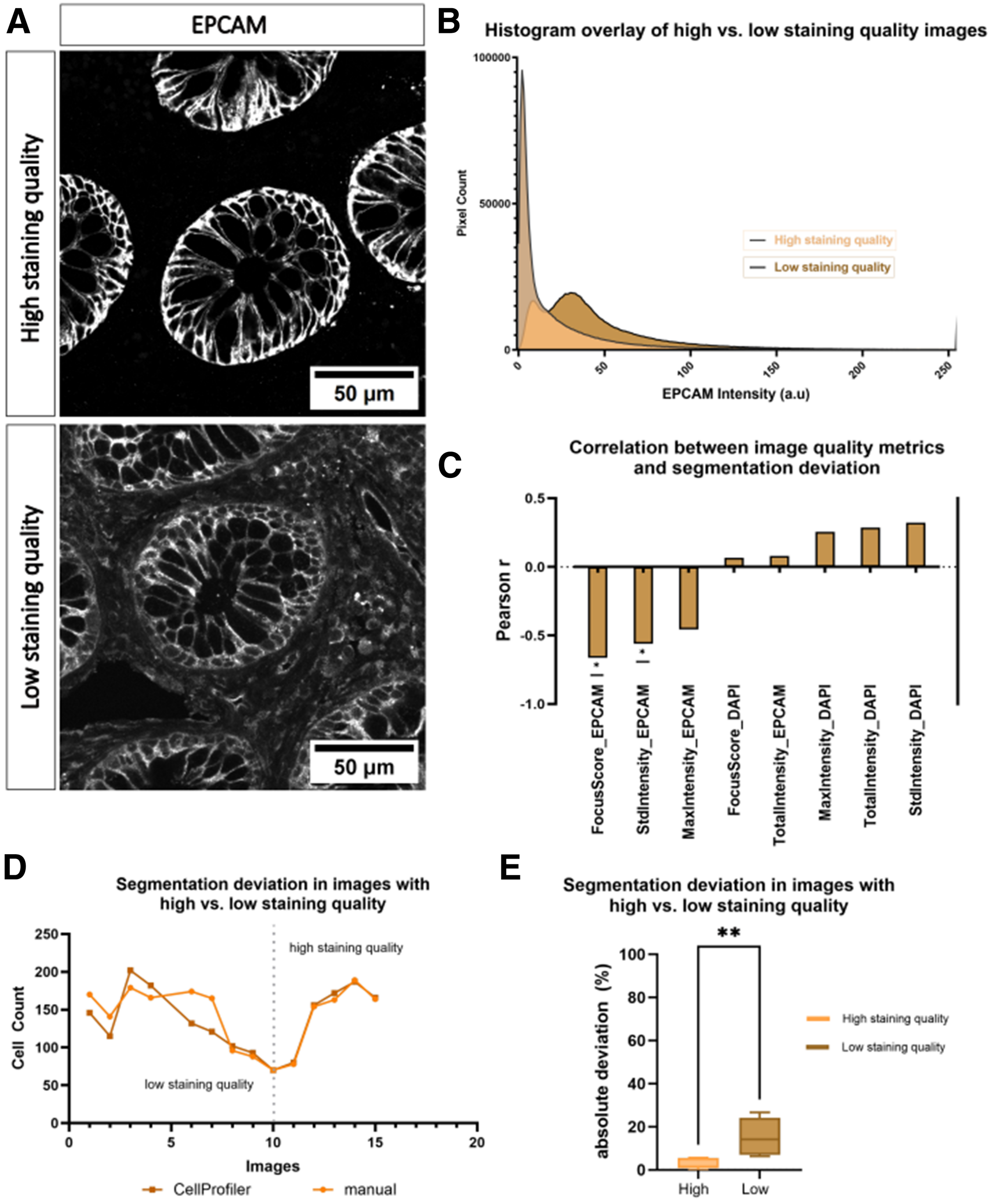

Figure 5. Impact of different image staining quality on segmentation performance. (*A*) Representative images examples showing high (*top*) and low (*bottom*) staining quality with the segmentation marker EpCAM. Scale bar represents 50 μm. (*B*) Overlay of histograms display the distribution of mean pixel intensities (EpCAM channel) across images categorized as either high or low staining quality. (*C*) Correlation analysis between CellProfiler image quality metrics and segmentation performance of the PCP pipeline. (*D*) Representative line plot comparing the number of segmented cells (automated vs manual). Images with low staining quality show greater divergence (10.38%) between manual and automated counts, whereas images with good staining quality show better agreement with a deviation of 2.47%. (*E*) Boxplot showing the percentage deviation between automated and manual segmentation, grouped by staining quality. Low-quality images exhibited significantly higher segmentation error compared with high-quality images. Groups were defined based on EpCAM staining quality assessed visually and confirmed by image quality metrics (n = 15 images analyzed). Following a Shapiro-Wilk normality test, Welch's *t*-tests were used for statistical comparison. Significance levels were indicated as follow: ns = not significant; $^*P < .05$; $^{**}P < .01$; $^{***}P < .001$; and $^{****}P < .0001$.

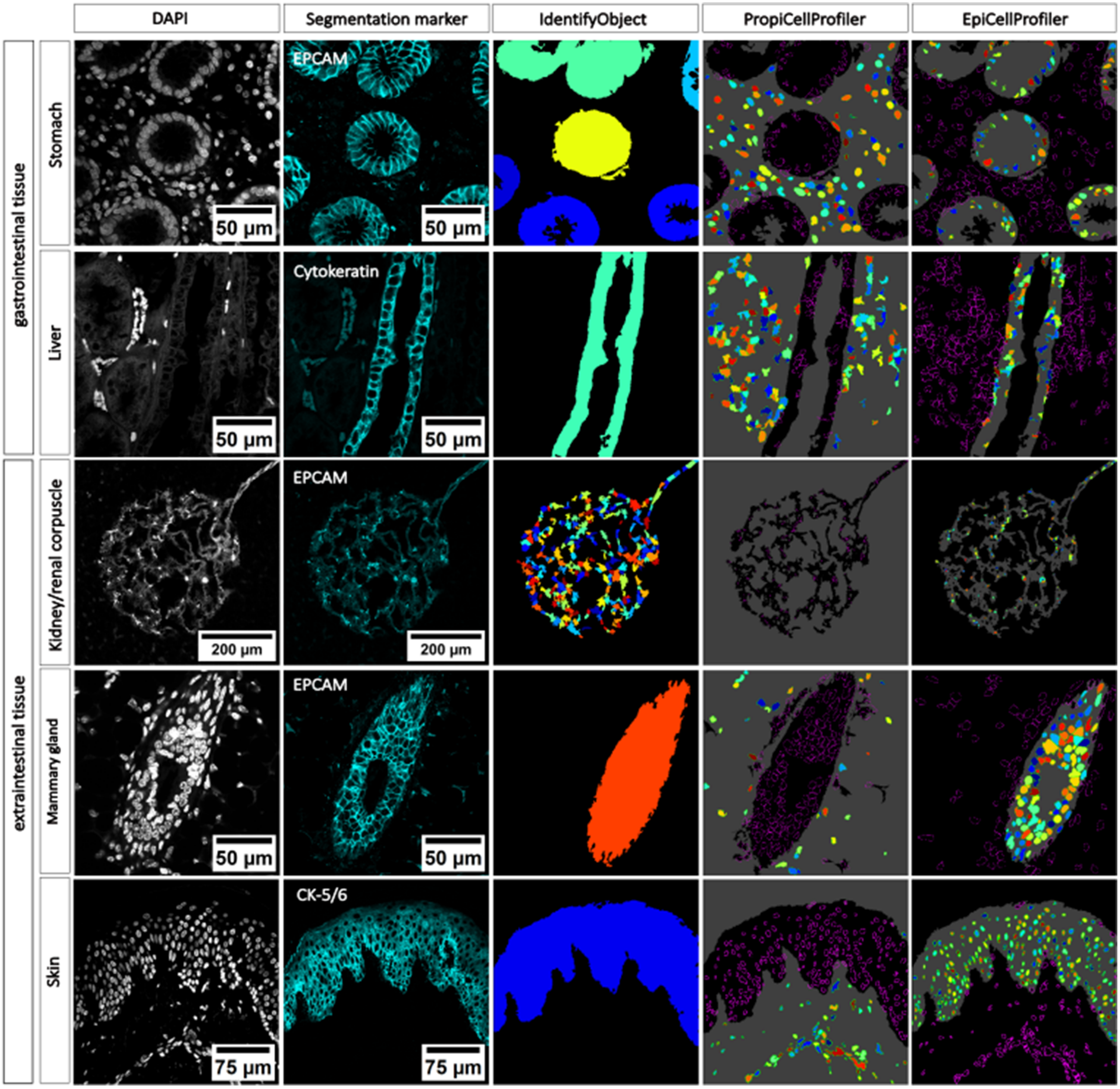

Figure 6. Evaluation of an expansion of automated cell segmentation across different human tissues. Representative immunofluorescence images and corresponding segmentations were reproduced in gastrointestinal (stomach and liver) and extraintestinal tissues (kidney, mammary gland, and skin) stained with the indicated antibodies. The pipeline achieved robust epithelial segmentations across all evaluated tissues and under the use of different primary antibodies. Stomach, liver, and mammary gland were acquired at 63×; kidney and skin at 20× magnification. Scale bars in μm are indicated.

our workflow. First, we applied the *FilterObjects* module into our pipelines to remove potential oversegmented cells that deviate from the mean area value by a factor of 3 (Figure 3*A*; *cyan-marked cell*). With that, few cells were removed within an image, but these incorrectly segmented cells were not included in the quantitative morphometric comparison. Secondly, we developed an automated quality assessment pipeline that assesses predictive features to identify low-quality images before data analysis. This combination of protocol optimization and image quality filtering improves the consistency of segmentation across tissue samples with potential varying levels of staining. Despite the variability in marker staining, strong correlations were observed between manual and automated cell counts, even in images with low EpCAM quality. This highlights the robustness of our segmentation approach and underscores the value of CellProfiler as a tool for quantitative image analysis. However, it is important to note that the current version does not distinguish between specific cell types within the lamina propria. Incorporating additional cell-type-specific markers in future applications could increase the usefulness of this workflow for targeted immunological studies.

We further elucidate the performance of our pipelines. With that, we compared the presented pipeline with another widely used image tool, namely ImageJ. We found that with an increasing number of spot counts per image, the differences between ImageJ and CellProfiler become greater. Concretely, this problem becomes relevant when the spot number exceeds 5000 per image. This finding may represent an important limitation when comparing data from different studies analysed with specific software tools. In our study, we included a diverse set of images with a considerable range in terms of 'observed limits of agreement' and 'degree of tissue coverage,' which may have contributed to this observation. However, this variability does not substantially affect the comparative outcomes between ImageJ and CellProfiler, as both software tools analyzed the same set of images under similar conditions. There are several limitations in image analysis software that may result in different spot counts. Different software may use different algorithms to identify spots, with diverging approaches to thresholding, noise reduction, and object recognition. Some programs may be more sensitive to detecting smaller or fainter spots, and the difference between sensitivity and specificity may result in unequal counts. It is important to understand the capabilities and settings of each software and try to standardize parameters between tools for future purposes.

CellProfiler has proven its versatility in numerous research studies, particularly in cell culture and high-throughput screening applications. However, its applicability to complex human tissue imaging has not been systematically explored. This work shows that our segmentation pipelines, originally developed to distinguish intestinal epithelial regions from the surrounding lamina propria, can be adapted to other human tissue types. The transferability was most successful in tissues with characteristic epithelial features, such as mammary gland, stomach, kidney, and skin, whereas in structurally homogenous tissues like the liver, EpCAM-based segmentation lacked biological relevance.

The aim of this study was to develop and present to the scientific community a robust analytical pipeline facilitating quantitative biological assessments in gastrointestinal tissues. Given the multifunctional nature of the G3BP1 protein, future research will need to delineate its specific roles in different cellular morphotypes. The current understanding of G3BP1 and its association with SGs in the context of chronic inflammatory diseases is still emerging. This study provides observations on the distribution and localization of G3BP1 within gastrointestinal cellular structures in patients with CD and UC without active inflammation. Future investigations are warranted to elucidate the functional dynamics of SGs in the setting of active IBD and to determine their potential role in the pathogenesis of the disease. The findings regarding the differences in nuclear morphology within the subset of patients that were, at the time of biopsy collection, in clinical, biochemical, and endoscopic remission, are of considerable interest. However, these initial findings were made in a rather small group of patients, so it is important that these results are confirmed in a larger study. In conclusion, we have presented 2 novel pipelines that facilitate high-throughput analyses and quantification of specific proteins of interest in tissues, cells, and even subcellular compartments. Additionally, we describe subtle alterations of nuclear morphology in patients with CD and UC in remission. Designed for adaptability and accessibility, the pipelines may be used and adapted by the wider scientific community, helping to promote research by facilitating more efficient and comprehensive investigations of cellular behavior and protein dynamics. Moreover, our findings contribute to the growing pool of knowledge on the role of SGs in chronic diseases. In our hands, CellProfiler proved as a useful tool for cellular and molecular investigations in IBD.

Materials and Methods

Patients and Biopsy Specimens

Colonic pinch biopsy specimens were obtained during endoscopic investigations from 10 patients with CD and UC in remission (no clinical symptoms, negative fecal calprotectin, normal appearance of the mucosa in endoscopy, histologic remission). Patients who did not meet clinical criteria for IBD were assigned to the control group. Endoscopic remission was determined, following previous guidelines[49,50] as having an eMayo score of either 0 or 1, or a Simple Endoscopic Score for Crohn's Disease (SES-CD) score of 2 or less. The use of colonic biopsies from patients with UC and CD in remission for the design of our pipelines was approved by the ethics committee of the Medical University Linz (EK-No. 1012/2025). To demonstrate transferability of the pipelines to other human tissues, an additional approval was obtained (EK-No. 1253/2025). Written informed consent was obtained from all patients prior to enrollment.

Immunofluorescence Staining and Confocal Imaging

Noninflamed colon samples from the rectum and sigmoid colon were frozen in liquid nitrogen and cut using Leica microtome (Leica Biosystems) at a thickness of 4 μm and transferred on superfrost microscope slides. Tissue sections were fixed with 4% paraformaldehyde for 15 minutes at room temperature (RT) followed by peroxidase block (Cedarlane Labs) for 10 minutes at RT. Immunofluorescence staining was carried out as previously described.[51] Briefly, prior to incubation with the primary antibody, tissue sections were washed 2 times in phosphate buffered saline with Tween 20 (PBST), and unspecific binding sites were blocked using a serum-free protein block for 20 minutes at RT (Protein Block, Serum-Free, Ready-To-Use; Dako Agilent Technologies). Blocking solution was discarded on tissue towels, and 100 μL of primary antibody diluted in Dako Real Antibody Diluent (Agilent Technologies) was added. Slides were incubated at 4°C overnight in a humidity chamber. Primary antibodies used were: rabbit anti-G3BP1 (1:200, HPA004052, Sigma Life Science), and goat anti-hEpCAM (1:200, AF960, R&D Systems). Additional human tissue specimens, obtained as residual material from the Department of Pathology,

Medical University Linz, were stained with mouse anti-Cytokeratin (1:200, M3515, Agilent Technologies) or rabbit anti-Cytokeratin 5/6 (1:200; MA5-33043, Invitrogen). Secondary antibodies used were Alexa Flour 647 anti-rabbit (IgG) (1:500; 406414, BioLegend) and Alexa Fluor 488 anti-goat (IgG) (1:500; A-11055; Invitrogen), or Alexa Fluor 555 anti-mouse (IgG) (1:500; A-21422; Invitrogen). Negative control tissue sections were incubated with Dako Real Antibody Diluent without primary antibody. After staining with respective secondary antibodies and thorough washing steps, tissue sections were mounted using ProLong Gold antifade reagent with DAPI (Thermo Fischer Scientific Inc). Microscopic slides were dried for 24 hours at RT in the dark before imaging on a confocal laser scanning microscope (STELLARIS 5, Leica Microsystem CMS GmbH) or a STED Facility Line microscope (Abberior). Acquisition parameters were the same for each quantified image set.

Equipment and Software

The following software packages were used: (1) LEICA software to work with acquired images (.lif files): LASX Office 1.4.5 downloaded from the following website and used to enhance contrast for representing images in Figures 7*A* and 8*A*: https://www.leica-microsystems.com/de/produkte/mikroskop-software/p/leica-las-x-ls/downloads/.

For quantitative image analysis using CellProfiler, raw images were exported directly as .tiff files from LASX Office. Note: If exported images were used for analysis, deactivating "scale bar" settings are necessary because graphics interfere with image structure detection. (2) Open-source CellProfiler image analysis software: CellProfiler (version 4.2.6 at time of publication), downloaded from the CellProfiler website (www.cellprofiler.org) and used on a MacBook Pro, macOS Ventura (2,3 GHz Quad-Core Intel Core i5), 8 GB, 2133 MHz. The automated and customized CellProfiler-based pipelines, used in this work, can be downloaded from the Supplementary Material.

The pipelines, together with example images and documentation, are openly available on GitHub:

PCP pipeline: https://github.com/HelenaHoedlmayr/PropiCellProfiler

ECP pipeline: https://github.com/HelenaHoedlmayr/EpiCellProfiler

Development of EPC Pipeline for Segmentation of IECs and G3BP1 Protein Distribution

The EPC pipeline has been developed to segment IECs and to analyze protein distribution of any target of interest. Each image used comprises 3 channels, one for nuclear staining (DAPI), one for the cell membrane staining (EpCAM), and a third for the key player in stress granule life cycle, G3BP1 (Figure 7*A*). First, images were imported into the CellProfiler software by the 'drag and drop' feature from the file manager tool. The detailed setup steps for starting a project are described elsewhere.[52] The workflow for the analyses begins with the design of a pipeline. There are different modules to process files, images, or objects, or to perform specific biological analyses. Most pipelines depend on the identification of objects. In CellProfiler, the objects to be segmented are referred to as primary, secondary, or tertiary objects. EpCAM staining was first used to delineate IEC boundaries within the *IdentifyPrimaryObject* module, and secondly to discard nonepithelial nuclear objects outside this specified region. The panels in Figure 7*B* represent some major steps of the pipeline's workflow: (I) segmentation of primary nuclei objects within an image; (II) masking the intestinal epithelium through the cyan channel; and (III) removing nonepithelial cells by reversing the foreground/background relationship of the mask; (IV) segmentation of the whole cell; and (V) subtracting the nucleus from the cell object to identify the cytoplasm region; (VI) identification of G3BP1 granules as primary object; (VII) More detailed view of G3BP1 granules identification from the same image. Panel number VIII shows the final segmentation of the cells (*gray*), nuclei (*cyan*), and G3BP1 punctate (*magenta*). Detailed information on the modules and settings used for cell segmentation in the ECP pipeline are provided in the Supplementary Materials (Supplementary Table 1). The modules *MeasureObjectIntensityDistribution*, *MeasureObjectSizeShape*, and *MeasureObjectIntensity* were used to quantify the size, area, and intensity distribution of G3BP1, focusing on its spatial distribution within IECs. For the *MeasureObjectIntensityDistribution*, we specified 3 rings within the defined cytoplasmic objects and excluded the nucleus region. Doing so, we measured the total intensity of G3BP1 at a given radius, namely in bin 1 (= innermost), bin 2 (= intermediate), and bin 3 (= outermost). In the final step, *ExportToSpreadsheet* was used to export measurements into separate files that could be opened in Excel or Numbers.

Development of an Automated PCP Pipeline for the Identification and Quantification of Immune-Related Cells in the Lamina Propria

The PCP pipeline was designed to automate the identification and quantification of cells in the lamina propria, thereby providing a more comprehensive understanding of the behavior of cells that participate actively in mucosal inflammation. Prior modules from the ECP for the identification of G3BP1 in intestinal tissue have been incorporated into this pipeline together with measurements of various nuclear morphometric parameters. Images were labeled with DAPI (*gray*) as nuclear staining, EpCAM (*cyan*) to outline the epithelium, and G3BP1 (*magenta*) as the protein of interest (Figure 8*A*). The panels in Figure 8*B* represent some major steps of the pipeline's workflow: (I) segmentation of primary nuclei objects within the whole image; (II) masking the intestinal epithelium through the cyan channel; and (III) discarding cells in that specified region; (IV) delineation of whole cell objects by a specified number of pixels; and (V) identification of G3BP1 spots. Panel (VI) shows the *FilterObject* to distinguish between G3BP1-positive and -negative objects. Panel number VII represents the final segmentation of all objects (cyan if G3BP1-positive and magenta if G3BP1-negative), and G3BP1

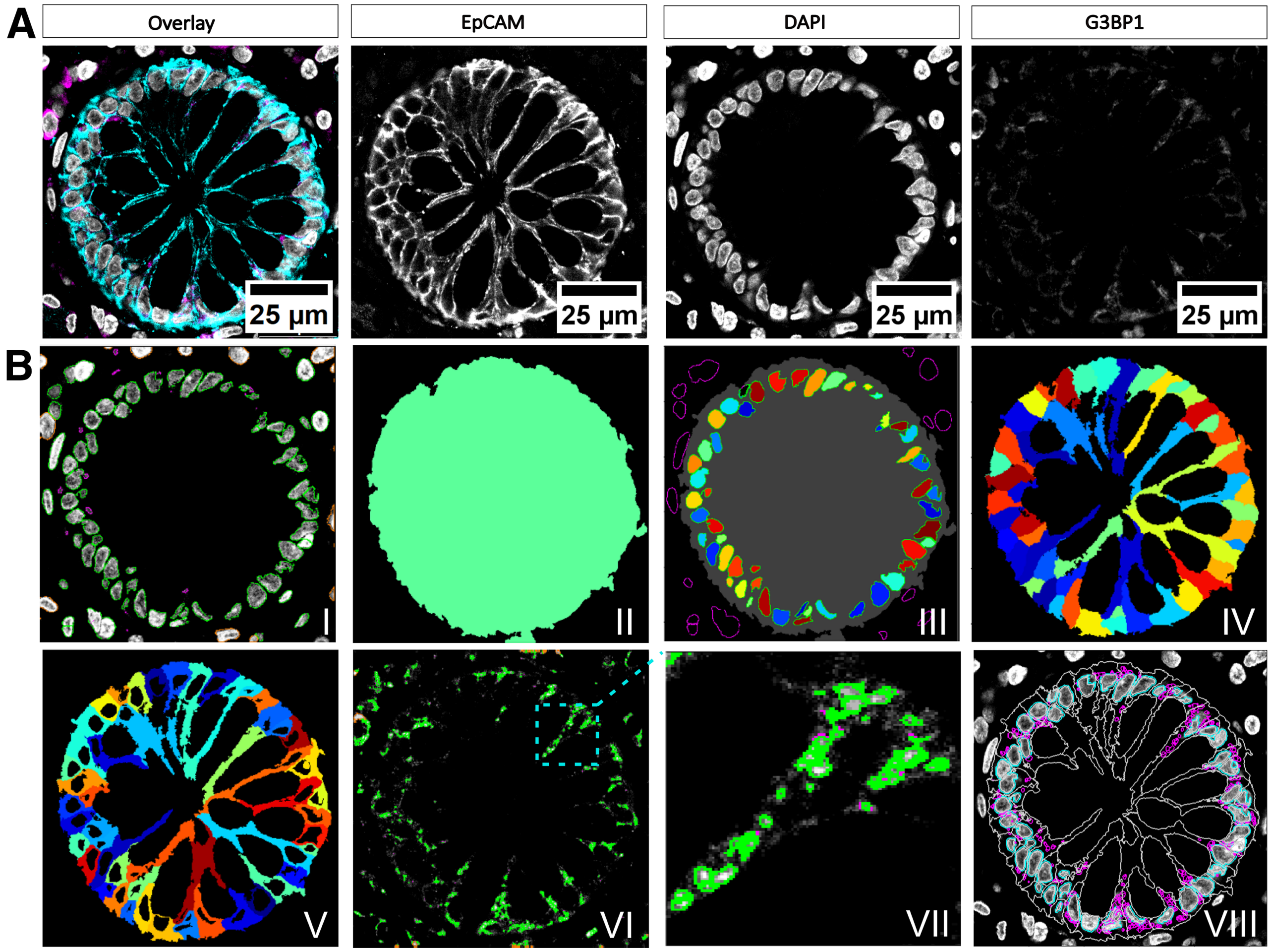

Figure 7. Laser-scanning confocal image of a human colonic crypt with the ECP workflow applied. (*A*) Representative laser-scanning confocal image, illustrating EpCAM immunoreactivity (*cyan*), DAPI (*gray*), and G3BP1 (*magenta*). Scale bars represent 25 μm as indicated. (*B*) Illustrative images of the pipeline's workflow to identify IECs within the EpCAM-stained crypt. The *IdentifyPrimaryObjects* module was initially applied to delineate nuclei (*I*) and to recognize the intestinal region in the image (*II*). The *MaskObjects* module was then implemented to remove the nuclei of cells that were not within the cryptical region (*III*). Subsequently, the *IdentifySecondaryObjects* module was used to define a cell object, utilizing the nuclei as a point of reference (*IV*). The *IdentifyTertiaryObjects* module was applied to define the cytoplasm, utilizing the nucleus as the primary object and the cell as the secondary object (*V*). *IdentifyPrimaryObjects* module was employed to identify G3BP1 spots (*VI*). *Image VII* illustrates a comprehensive delineation of G3BP1 granules. The combination of all final objects permits the identification of individual nuclei (*cyan*), cytoplasm outer lines (*gray*), and G3BP1 spots (*magenta*) within a final image (*VIII*).

granules (*yellow*). Cells with at least 2 granules were evaluated as positive (VIII). *MeasureObjectIntensity* and *MeasureObjectSizeShape* modules were used to perform a comparative analyses of different nuclear morphology parameters and SG abundance between control and patients with UC and CD in remission. Again, *ExportToSpreadsheet* was used in the last step to export measurements into separate files that could be opened in Excel or Numbers. Detailed settings information for all used modules can be seen in the Supplementary Material (Supplementary Table 2).

STED Microscopy

Intestinal sections were stained for detection of G3BP1 as previously described. Primary antibody included rabbit *anti*-G3BP1 (1:200, HPA004052, Sigma Life Science) in combination with STAR RED labelled goat-anti-rabbit (STED-1002-500UG, Abberior) For STED, cells were acquired using a 60×, NA 1.4 oil objective by confocal and STED microscopy employing an Abberior STED Facility Line Microscope instrument (Aberrior). STAR RED-labeled probes for G3BP1 detection were excited at 640 nm, and a pulsed depletion laser at 775 nm was used with a typical maximum power of 20%. For the nucleus, DAPI-labeled probes were excited using 405 nm. Images were processed using Huygens software (Scientific Volume Imaging; http://svi.nl) and Icy.[53]

Manual Cell Counting and Speckle Scoring with Other Software

To validate the data generated by CellProfiler, colonic epithelial cell counts and cells within the lamina propria were determined manually under blindfolded conditions, and results were written to Excel files. Particle counts were

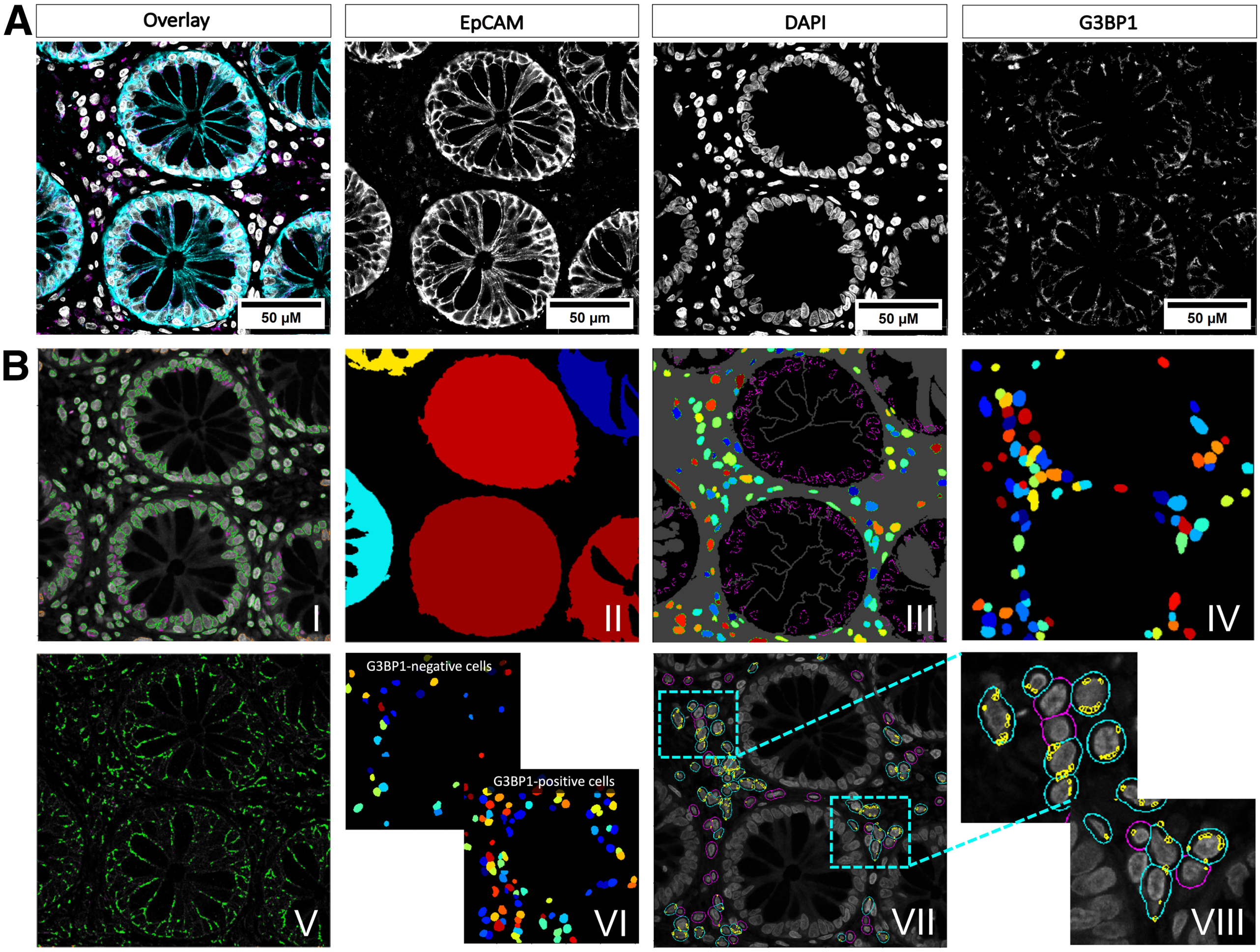

Figure 8. Laser-scanning confocal images of human colon cross sections with the PCP workflow applied. (*A*) Representative laser-scanning confocal images, stained with EpCAM (*cyan*), DAPI (*gray*), and G3BP1 (*magenta*). Scale bars represent 50 μm as indicated. (*B*) Illustrative images of the pipeline's workflow to identify cells within the lamina propria. The *IdentifyPrimaryObjects* module was initially applied to delineate nuclei (*I*) and to recognize the intestinal feature within the whole image (*II*). The *MaskObjects* module was then implemented to remove the nuclei of cells that were within the specified intestinal region (*III*). Subsequently, the *IdentifySecondaryObjects* module was used to define a cell object by a specific set of pixels from the nucleus as starting point (*IV*). To identify G3BP1 spots, the *IdentifyPrimaryObjects* module was employed once more on the G3BP1 immunoreactivity channel (*V*). *Image VI* illustrates a comprehensive delineation of G3BP1-positive and -negative cells. The combination of all final objects permits the monitoring of individual cells (*cyan* for G3BP1-positive and *magenta* for G3BP1-negative), and G3BP1 spots (*yellow*) within a final image (*VII* and *VIII*).

further analyzed using ImageJ version 2.3.0[54] with equal thresholding method 'Otsu' and similar pixel settings as used in CellProfiler for filtering particle size.

Statistical Analysis

A total of 5 patient samples were analyzed per group (control, CD, and UC in remission), with 4 images quantified per patient. Comparison between the control group and the CD and UC in remission group was performed by Mann-Whitney and Kruskal-Wallis tests as data were not normally distributed. Differences between multiple groups were analyzed using 1-way analysis of variance (ANOVA) with Kruskal-Wallis tests combined with Dunn post-tests for nonparametric data. Box plots show the data as median (central line) and minimum to maximum values (whiskers). Pearson's correlation between manual counting vs CellProfiler and Bland-Altman Plot for ImageJ software comparison was calculated and illustrated using Graph-Pad Prism 10.3.0 (GraphPad Software). Significance levels were indicated as follows: ns = not significant; $*P < .05$; $**P < .01$; $***P < .001$; and $****P < .0001$.

Supplementary Material

Note: To access the supplementary material accompanying this article, visit the full text version at https://doi.org/10.1016/j.jcmgh.2025.101680.

References

1. Turner JR. Intestinal mucosal barrier function in health and disease. Nat Rev Immunol 2009;9:799–809.

2. Artis D. Epithelial-cell recognition of commensal bacteria and maintenance of immune homeostasis in the gut. Nat Rev Immunol 2008;8:411–420.
3. Su L, Shen L, Clayburgh DR, et al. Targeted epithelial tight junction dysfunction causes immune activation and contributes to development of experimental colitis. Gastroenterology 2009;136:551–563.
4. Wallace KL, Zheng LB, Kanazawa Y, Shih DQ. Immunopathology of inflammatory bowel disease. World J Gastroenterol 2014;20:6–21.
5. Sanz G, Manuel Martínez-Aranda L, Tesch PA, et al. Muscle2View, a CellProfiler pipeline for detection of the capillary-to-muscle fiber interface and high-content quantification of fiber type-specific histology. J Appl Physiol 2019;127:1698–1709.
6. Ouzéna B, Tay TL. Automated cell counting of macrophages in situ. In: Mass E, ed. Tissue-Resident Macrophages: Methods and Protocols. New York, NY: Springer US, 2024:505–518.
7. Laan SNJ, Dirven RJ, Bürgisser PE, et al. Automated segmentation and quantitative analysis of organelle morphology, localization and content using CellProfiler. PLoS One 2023;18:e0278009.
8. Wills JW, Robertson J, Summers HD, et al. Image-based cell profiling enables quantitative tissue microscopy in gastroenterology. Cytometry Part A 2020; 97:1222–1237.
9. Carpenter AE, Jones TR, Lamprecht MR, et al. CellProfiler: image analysis software for identifying and quantifying cell phenotypes. Genome Biol 2006;7:R100.
10. Skinner BM, Johnson EEP. Nuclear morphologies: their diversity and functional relevance. Chromosoma 2017; 126:195–212.
11. Mitsui' Y, Schneider EL. Increased nuclear sizes in senescent human diploid fibroblast cultures. Exp Cell Res 1976;100:147–152.
12. Chen JH, Ozanne SE. Deep senescent human fibroblasts show diminished DNA damage foci but retain checkpoint capacity to oxidative stress. FEBS Lett 2006; 580:6669–6673.
13. Tourrière H, Gallouzi IE, Chebli K, et al. RasGAP-associated endoribonuclease G3BP: selective RNA degradation and phosphorylation-dependent localization. Mol Cell Biol 2001;21:7747–7760.
14. Irvine K, Stirling R, Hume D, Kennedy D. Rasputin, more promiscuous than ever: a review of G3BP. Int J Dev Biol 2004;48:1065–1077.
15. Yang P, Mathieu C, Kolaitis RM, et al. G3BP1 is a tunable switch that triggers phase separation to assemble stress granules. Cell 2020;181:325–345.e28.
16. Tourrière H, Chebli K, Zekri L, et al. The RasGAP-associated endoribonuclease G3BP mediates stress granule assembly. J Cell Biol 2023;222: e200212128072023new.
17. Burd CG, Dreyfusst G. Conserved structures and diversity of functions of RNA-binding proteins. Science 1994;265:615–621.
18. Kang W, Wang Y, Yang W, et al. Research progress on the structure and function of G3BP. Front Immunol 2021;12:a032813.
19. Mao C, Wang X, Liu Y, et al. G3BP1-interacting lncRNA promotes ferroptosis and apoptosis in cancer via nuclear sequestration of p53. Cancer Res 2018; 78:3484–3496.
20. Kim MM, Wiederschain D, Kennedy D, et al. Modulation of p53 and MDM2 activity by novel interaction with Ras-GAP binding proteins (G3BP). Oncogene 2007;26:4209–4215.
21. Mowat AM, Agace WW. Regional specialization within the intestinal immune system. Nat Rev Immunol 2014; 14:667–685.
22. Ivanov P, Kedersha N, Anderson P. Stress granules and processing bodies in translational control. Cold Spring Harb Perspect Biol 2019;11:a032813.
23. Riggs CL, Kedersha N, Ivanov P, Anderson P. Mammalian stress granules and P bodies at a glance. J Cell Sci 2020;133:jcs242487.
24. Ramaswami M, Taylor JP, Parker R. Altered "Ribostasis" RNA-protein granule formation or persistence in the development of degenerative disorders. Cell 2013;154.
25. Zhou H, Luo J, Mou K, et al. Stress granules: functions and mechanisms in cancer. Cell Biosci 2023;13:86.
26. Cadena Sandoval M, Heberle AM, Rehbein U, et al. mTORC1 crosstalk with stress granules in aging and age-related diseases. Front Aging 2021;2:761333.
27. Guan Y, Wang Y, Fu X, et al. Multiple functions of stress granules in viral infection at a glance. Front Microbiol 2023;14.
28. Onomoto K, Yoneyama M, Fung G, et al. Antiviral innate immunity and stress granule responses. Trends Immunol 2014;35:420–428.
29. Hu S, Claud EC, Musch MW, Chang EB. Stress granule formation mediates the inhibition of colonic Hsp70 translation by interferon-and tumor necrosis factor. Am J Physiol Gastrointest Liver Physiol 2010;298:481–492.
30. Curdy N, Lanvin O, Cerapio JP, et al. The proteome and transcriptome of stress granules and P bodies during human T lymphocyte activation. Cell Rep 2023;42:112211.
31. Huang Y, Feng Y, Cui L, et al. Autophagy-related LC3 accumulation interacted directly with LIR containing RIPK1 and RIPK3, stimulating necroptosis in hypoxic cardiomyocytes. Front Cell Dev Biol 2021;9:679637.
32. Shim MS, Nettesheim A, Hirt J, Liton PB. The autophagic protein LC3 translocates to the nucleus and localizes in the nucleolus associated to NUFIP1 in response to cyclic mechanical stress. Autophagy 2020;16:1248–1261.
33. Rosenberg L, Lawlor GO, Zenlea T, et al. Predictors of endoscopic inflammation in patients with ulcerative colitis in clinical remission. Inflamm Bowel Dis 2013; 19:779–784.
34. Zhang CH, Wang JX, Cai ML, et al. The roles and mechanisms of G3BP1 in tumour promotion. J Drug Target 2019;27:300–305.
35. Li Y, Wang J, Zhong S, et al. Overexpression of G3BP1 facilitates the progression of colon cancer by activating β-catenin signaling. Mol Med Rep 2020;22:4403–4411.
36. Xiong R, Gao JL, Yin T. G3BP1 activates the TGF-β/Smad signaling pathway to promote gastric cancer. Onco Targets Ther 2019;12:7149–7156.
37. Min L, Ruan Y, Shen Z, et al. Overexpression of Ras-GTPase-activating protein SH3 domain-binding protein

1 correlates with poor prognosis in gastric cancer patients. Histopathology 2015;67:677–688.
38. Zheng H, Zhan Y, Zhang Y, et al. Elevated expression of G3BP1 associates with YB1 and p-AKT and predicts poor prognosis in nonsmall cell lung cancer patients after surgical resection. Cancer Med 2019;8:6894–6903.
39. Eaden JA, Abrams KR, Mayberry JF. The risk of colorectal cancer in ulcerative colitis: a meta-analysis. Gut 2001;48:526–535.
40. Jess T, Gamborg M, Matzen P, et al. Increased risk of intestinal cancer in Crohn's disease: a meta-analysis of population-based cohort studies. Am J Gastroenterol 2005;100:2724–2729.
41. Chow KH, Factor RE, Ullman KS. The nuclear envelope environment and its cancer connections. Nat Rev Cancer 2012;12:196–209.
42. de Nader GPF, Agüera-Gonzalez S, Routet F, et al. Compromised nuclear envelope integrity drives TREX1-dependent DNA damage and tumor cell invasion. Cell 2021;184:5230–5246.e22.
43. Bell ES, Lammerding J. Causes and consequences of nuclear envelope alterations in tumour progression. Eur J Cell Biol 2016;95:449–464.
44. Prissette M, Fury W, Koss M, et al. Disruption of nuclear envelope integrity as a possible initiating event in tauopathies. Cell Rep 2022;40:111249.
45. Zheng L, Duan SL, Wen XL, Dai YC. Molecular regulation after mucosal injury and regeneration in ulcerative colitis. Front Mol Biosci 2022;9:996057.
46. Choi J, Augenlicht LH. Intestinal stem cells: guardians of homeostasis in health and aging amid environmental challenges. Exp Mol Med 2024;56:495–500.
47. Campisi J, D'Adda Di Fagagna F. Cellular senescence: when bad things happen to good cells. Nat Rev Mol Cell Biol 2007;8:729–740.
48. Omer A, Barrera MC, Moran JL, et al. G3BP1 controls the senescence-associated secretome and its impact on cancer progression. Nat Commun 2020;11:4979.
49. Daperno M, D'Haens G, Van Assche G, et al. Development and validation of a new, simplified endoscopic activity score for Crohn's disease: the SES-CD. Gastrointest Endosc 2004;60:505–512.
50. Peyrin-Biroulet L, Sandborn W, Sands BE, et al. Selecting therapeutic targets in inflammatory bowel disease (STRIDE): determining therapeutic goals for treat-to-target. Am J Gastroenterol 2015;110:1324–1338.
51. Zaqout S, Becker LL, Kaindl AM. Immunofluorescence staining of paraffin sections step by step. Front Neuroanat 2020;14:582218.
52. Ward AO, Janbandhu V, Chapman G, et al. An image analysis protocol using CellProfiler for automated quantification of post-ischemic cardiac parameters. STAR Protoc 2022;3:101097.
53. De Chaumont F, Dallongeville S, Chenouard N, et al. Icy: an open bioimage informatics platform for extended reproducible research. Nat Methods 2012;9:690–696.
54. Schindelin J, Arganda-Carreras I, Frise E, et al. Fiji: an open-source platform for biological-image analysis. Nat Methods 2012;9:676–682.

Received April 29, 2025. Accepted November 8, 2025.

Correspondence
Address correspondence to: Alexander R. Moschen, PhD, Department of Internal Medicine 2 (Gastroenterology and Hepatology, Endocrinology and Metabolism, Nephrology, Rheumatology), Faculty of Medicine, Johannes Kepler University Linz, Krankenhausstraße 5, Linz 4020, Austria. e-mail: alexander.moschen@jku.at.

CRediT Authorship Contributions

Helena Hödlmayr (Formal analysis: Lead; Investigation: Lead; Methodology: Equal; Software: Lead; Validation: Lead; Visualization: Lead; Writing – original draft: Lead)

Christina Watschinger (Conceptualization: Equal; Methodology: Equal; Project administration: Lead)

Gerald Karl Wallner (Data curation: Supporting)

Sabine Knipp (Resources: Equal; Writing – review & editing: Equal)

Arndt Rohwedder (Methodology: Equal; Resources: Equal; Software: Equal)

Regina Prommer (Data curation: Lead)

Rupert Langer (Resources: Supporting)

Alexander Rupert Moschen, PhD (Conceptualization: Supporting; Supervision: Lead; Writing – original draft: Supporting; Writing – review & editing: Lead)

Conflicts of interest
The authors disclose no conflicts.

Data Availability
The data that support the findings of this study are available from the corresponding author upon reasonable request. The pipelines, including example images and documentation, are openly accessible on GitHub.

Cellular and Molecular Gastroenterology and Hepatology, Volume *20*

Supplemental information

Design of CellProfiler-Based Pipelines Enabling the Attribution of Molecular Stress Markers to Specific Tissue and Subcellular Compartments of the Colonic Mucosa

Helena Hödlmayr, Christina Watschinger, Gerald K. Wallner, Sabine Knipp, Arndt Rohwedder, Regina Prommer, Rupert Langer, and Alexander R. Moschen

Key,Value
CellProfiler_Version,4.2.7
ChannelType_DAPI,Grayscale
ChannelType_EPCAM,Grayscale
ChannelType_G3BP1,Grayscale
Metadata_Tags,"[""ImageNumber""]"
Pipeline_Pipeline,"CellProfiler Pipeline: http://www.cellprofiler.org
Version:5
DateRevision:427
GitHash:
ModuleCount:21
HasImagePlaneDetails:False

Images:[module_num:1|svn_version:'Unknown'|variable_revision_number:2|show_window:False|notes:['To begin creating your project, use the Images module to compile a list of files and/or folders that you want to analyze. You can also specify a set of rules to include only the desired files in your selected folders.']|batch_state:array([], dtype=uint8)|enabled:True|wants_pause:False]

:
Filter images?:Images only
Select the rule criteria:and (extension does isimage) (directory doesnot containregexp ""[\\\\/]\\."")

Metadata:[module_num:2|svn_version:'Unknown'|variable_revision_number:6|show_window:False|notes:['The Metadata module optionally allows you to extract information describing your images (i.e, metadata) which will be stored along with your measurements. This information can be contained in the file name and/or location, or in an external file.']|batch_state:array([], dtype=uint8)|enabled:True|wants_pause:False]
Extract metadata?:No
Metadata data type:Text
Metadata types:{}
Extraction method count:1
Metadata extraction method:Extract from file/folder names
Metadata source:File name

Regular expression to extract from file name:^(?P<Plate>.*)_(?P<Well>[A-P][0-9]{2})_s(?P<Site>[0-9])_w(?P<ChannelNumber>[0-9])
Regular expression to extract from folder name:(?P<Date>[0-9]{4}_[0-9]{2}_[0-9]{2})$
Extract metadata from:All images
Select the filtering criteria:and (file does contain """")
Metadata file location:Elsewhere...|
Match file and image metadata:[]
Use case insensitive matching?:No
Metadata file name:None
Does cached metadata exist?:No

NamesAndTypes:[module_num:3|svn_version:'Unknown'|variable_revision_number:8|show_window:False|notes:['The NamesAndTypes module allows you to assign a meaningful name to each image by which other modules will refer to it.']|batch_state:array([], dtype=uint8)|enabled:True|wants_pause:False]
Assign a name to:Images matching rules
Select the image type:Grayscale image
Name to assign these images:DNA
Match metadata:[]
Image set matching method:Order
Set intensity range from:Image metadata
Assignments count:3
Single images count:0
Maximum intensity:255.0
Process as 3D?:No
Relative pixel spacing in X:1.0
Relative pixel spacing in Y:1.0
Relative pixel spacing in Z:1.0
Select the rule criteria:and (file does containregexp ""ch01"")
Name to assign these images:DAPI
Name to assign these objects:Cell
Select the image type:Grayscale image
Set intensity range from:Image metadata
Maximum intensity:255.0
Select the rule criteria:and (file does containregexp ""ch00"")

Name to assign these images:EPCAM
Name to assign these objects:Cell
Select the image type:Grayscale image
Set intensity range from:Image metadata
Maximum intensity:255.0
Select the rule criteria:and (file does containregexp ""ch02"")
Name to assign these images:G3BP1
Name to assign these objects:Cell
Select the image type:Grayscale image
Set intensity range from:Image metadata
Maximum intensity:255.0

Groups:[module_num:4|svn_version:'Unknown'|variable_revision_number:2|show_window:False|notes:['The Groups module optionally allows you to split your list of images into image subsets (groups) which will be processed independently of each other. Examples of groupings include screening batches, microtiter plates, time-lapse movies, etc.']|batch_state:array([], dtype=uint8)|enabled:True|wants_pause:False]
Do you want to group your images?:No
grouping metadata count:1
Metadata category:None

CorrectIlluminationApply:[module_num:5|svn_version:'Unknown'|variable_revision_number:5|show_window:False|notes:['Accurate segmentation of nucleus objects is crucial for subsequent steps and is highly dependent on the effectiveness of the staining process. Substracting EpCAM from the DAPI channel increases the distance between adjacnt nuclei, improving segmentation in the IdentifyPrimaryObjects module. ']|batch_state:array([], dtype=uint8)|enabled:True|wants_pause:False]
Select the input image:DAPI
Name the output image:CorrDAPI
Select the illumination function:EPCAM
Select how the illumination function is applied:Subtract
Set output image values less than 0 equal to 0?:Yes
Set output image values greater than 1 equal to 1?:Yes

IdentifyPrimaryObjects:[module_num:6|svn_version:'Unknown'|variable_revision_number:15|show_window:False|notes:['Over- or under segmentation is common, particuarly in complex tissues. To minimize this, it is recommended to acquire high-quality images. However, it should be noted that approx. 10% error rate per image can be expected.']|batch_state:array([], dtype=uint8)|enabled:True|wants_pause:False]
Select the input image:CorrDAPI
Name the primary objects to be identified:IdentifyNucleiObjects
Typical diameter of objects, in pixel units (Min,Max):15,75
Discard objects outside the diameter range?:Yes
Discard objects touching the border of the image?:Yes
Method to distinguish clumped objects:Shape
Method to draw dividing lines between clumped objects:Shape
Size of smoothing filter:10
Suppress local maxima that are closer than this minimum allowed distance:5
Speed up by using lower-resolution image to find local maxima?:Yes
Fill holes in identified objects?:After both thresholding and declumping
Automatically calculate size of smoothing filter for declumping?:Yes
Automatically calculate minimum allowed distance between local maxima?:Yes
Handling of objects if excessive number of objects identified:Continue
Maximum number of objects:500
Use advanced settings?:Yes
Threshold setting version:12
Threshold strategy:Global
Thresholding method:Minimum Cross-Entropy
Threshold smoothing scale:1.3488
Threshold correction factor:1.0
Lower and upper bounds on threshold:0.1,1.0
Manual threshold:0.0
Select the measurement to threshold with:None
Two-class or three-class thresholding?:Two classes
Log transform before thresholding?:No
Assign pixels in the middle intensity class to the foreground or the background?:Foreground
Size of adaptive window:50
Lower outlier fraction:0.05

Upper outlier fraction:0.05
Averaging method:Mean
Variance method:Standard deviation
# of deviations:2.0
Thresholding method:Minimum Cross-Entropy

IdentifyPrimaryObjects:[module_num:7|svn_version:'Unknown'|variable_revision_number:15|show_window:False|notes:['The IdentifyPrimaryObject module was chosen for segmenting the intestinal region due to its flexibility compared to creating a mask. EpCAm was occasionally found in the lamina propria. While there are alternative methods for filtering out specific regions in CellProfiler, this option yielded the best results four our images and region. ']|batch_state:array([], dtype=uint8)|enabled:True|wants_pause:False]
Select the input image:EPCAM
Name the primary objects to be identified:IdentifyIntestine
Typical diameter of objects, in pixel units (Min,Max):40,900
Discard objects outside the diameter range?:Yes
Discard objects touching the border of the image?:No
Method to distinguish clumped objects:None
Method to draw dividing lines between clumped objects:Shape
Size of smoothing filter:10
Suppress local maxima that are closer than this minimum allowed distance:7.0
Speed up by using lower-resolution image to find local maxima?:Yes
Fill holes in identified objects?:After declumping only
Automatically calculate size of smoothing filter for declumping?:Yes
Automatically calculate minimum allowed distance between local maxima?:Yes
Handling of objects if excessive number of objects identified:Continue
Maximum number of objects:500
Use advanced settings?:Yes
Threshold setting version:12
Threshold strategy:Global
Thresholding method:Minimum Cross-Entropy
Threshold smoothing scale:1.3488
Threshold correction factor:0.70
Lower and upper bounds on threshold:0.01,1.0
Manual threshold:0.0

Select the measurement to threshold with:None
Two-class or three-class thresholding?:Two classes
Log transform before thresholding?:No
Assign pixels in the middle intensity class to the foreground or the background?:Foreground
Size of adaptive window:50
Lower outlier fraction:0.05
Upper outlier fraction:0.05
Averaging method:Mean
Variance method:Standard deviation
# of deviations:2.0
Thresholding method:Minimum Cross-Entropy

MaskObjects:[module_num:8|svn_version:'Unknown'|variable_revision_number:3|show_window:False|notes:['First, the previous specified intestinal region was used for masking. Second, objects that are outside this region are removed, depending on overlap. ']|batch_state:array([], dtype=uint8)|enabled:True|wants_pause:False]
Select objects to be masked:IdentifyNucleiObjects
Name the masked objects:Nuclei
Mask using a region defined by other objects or by binary image?:Objects
Select the masking object:IdentifyIntestine
Select the masking image:None
Handling of objects that are partially masked:Remove
Fraction of object that must overlap:0.5
Numbering of resulting objects:Renumber
Invert the mask?:No

IdentifySecondaryObjects:[module_num:9|svn_version:'Unknown'|variable_revision_number:10|show_window:True|notes:['The nuclei objects are now used to identify whole cells. This is not a straightforward process for intestinal epithelial cells, as Goblet or Paneth cells have a large cytoplasm due to their secretory tasks. Unfortunately, we have not found a way to segment this region in secretory cells. It would be interestingto explore markers for specific segmentation of, for example, goblet cells. ', '', '']|batch_state:array([], dtype=uint8)|enabled:True|wants_pause:False]
Select the input objects:Nuclei
Name the objects to be identified:IdentifyCellObjects
Select the method to identify the secondary objects:Distance - N

Select the input image:EPCAM
Number of pixels by which to expand the primary objects:10
Regularization factor:0.05
Discard secondary objects touching the border of the image?:No
Discard the associated primary objects?:No
Name the new primary objects:FilteredNuclei
Fill holes in identified objects?:Yes
Threshold setting version:12
Threshold strategy:Global
Thresholding method:Minimum Cross-Entropy
Threshold smoothing scale:1.0
Threshold correction factor:1.0
Lower and upper bounds on threshold:0.01,1.0
Manual threshold:0.0
Select the measurement to threshold with:None
Two-class or three-class thresholding?:Two classes
Log transform before thresholding?:No
Assign pixels in the middle intensity class to the foreground or the background?:Foreground
Size of adaptive window:50
Lower outlier fraction:0.05
Upper outlier fraction:0.05
Averaging method:Mean
Variance method:Standard deviation
# of deviations:2.0
Thresholding method:Minimum Cross-Entropy

IdentifyTertiaryObjects:[module_num:10|svn_version:'Unknown'|variable_revision_number:3|show_window:True|notes:['Here, cytoplasm of cells are defined. ']|batch_state:array([], dtype=uint8)|enabled:True|wants_pause:False]
Select the larger identified objects:IdentifyCellObjects
Select the smaller identified objects:Nuclei
Name the tertiary objects to be identified:Cytoplasm
Shrink smaller object prior to subtraction?:Yes

EnhanceOrSuppressFeatures:[module_num:11|svn_version:'Unknown'|variable_revision_number:7|show_window:True|notes:['Enhancing features such as speckles can increase the contrast between the objects of interest and the backgroud, making it easier to distinguish specific features within the image. Also, algorithms can detect more accurately, segment, and classify objects. ', '', '']|batch_state:array([], dtype=uint8)|enabled:True|wants_pause:False]
    Select the input image:G3BP1
    Name the output image:EnhanceOrSuppressFeatures
    Select the operation:Enhance
    Feature size:10
    Feature type:Speckles
    Range of hole sizes:1,10
    Smoothing scale:2.0
    Shear angle:0.0
    Decay:0.95
    Enhancement method:Line structures
    Speed and accuracy:Fast
    Rescale result image:Yes

IdentifyPrimaryObjects:[module_num:12|svn_version:'Unknown'|variable_revision_number:15|show_window:True|notes:[]|batch_state:array([], dtype=uint8)|enabled:True|wants_pause:False]
    Select the input image:EnhanceOrSuppressFeatures
    Name the primary objects to be identified:IdentifyG3BP1Objects
    Typical diameter of objects, in pixel units (Min,Max):2,30
    Discard objects outside the diameter range?:Yes
    Discard objects touching the border of the image?:Yes
    Method to distinguish clumped objects:Intensity
    Method to draw dividing lines between clumped objects:Intensity
    Size of smoothing filter:10
    Suppress local maxima that are closer than this minimum allowed distance:7.0
    Speed up by using lower-resolution image to find local maxima?:Yes
    Fill holes in identified objects?:After both thresholding and declumping
    Automatically calculate size of smoothing filter for declumping?:Yes
    Automatically calculate minimum allowed distance between local maxima?:Yes
    Handling of objects if excessive number of objects identified:Continue

Maximum number of objects:500
Use advanced settings?:Yes
Threshold setting version:12
Threshold strategy:Global
Thresholding method:Minimum Cross-Entropy
Threshold smoothing scale:2
Threshold correction factor:1.0
Lower and upper bounds on threshold:0.04,1.0
Manual threshold:0.0
Select the measurement to threshold with:None
Two-class or three-class thresholding?:Two classes
Log transform before thresholding?:No
Assign pixels in the middle intensity class to the foreground or the background?:Foreground
Size of adaptive window:50
Lower outlier fraction:0.05
Upper outlier fraction:0.05
Averaging method:Mean
Variance method:Standard deviation
# of deviations:2.0
Thresholding method:Minimum Cross-Entropy

RelateObjects:[module_num:13|svn_version:'Unknown'|variable_revision_number:5|show_window:False|notes:['Here, we relate previous identifiy G3BP1 spots with cells, cytoplasm or nucleus (in seperate steps) to compare intensity, respectively. ', '', 'Note: The output later is extensive, so take the time to labbel objects appropriate, to retrieve desired measurements. ']|batch_state:array([], dtype=uint8)|enabled:True|wants_pause:False]
Parent objects:IdentifyCellObjects
Child objects:IdentifyG3BP1Objects
Calculate child-parent distances?:None
Calculate per-parent means for all child measurements?:No
Calculate distances to other parents?:No
Do you want to save the children with parents as a new object set?:Yes
Name the output object:RelateG3BP1ToCellObjects
Parent name:None

RelateObjects:[module_num:14|svn_version:'Unknown'|variable_revision_number:5|show_window:False|notes:[]|batch_state:array([], dtype=uint8)|enabled:True|wants_pause:False]
    Parent objects:Cytoplasm
    Child objects:IdentifyG3BP1Objects
    Calculate child-parent distances?:None
    Calculate per-parent means for all child measurements?:Yes
    Calculate distances to other parents?:No
    Do you want to save the children with parents as a new object set?:Yes
    Name the output object:RelateG3BP1ToCytoplasmObjects
    Parent name:None

RelateObjects:[module_num:15|svn_version:'Unknown'|variable_revision_number:5|show_window:False|notes:[]|batch_state:array([], dtype=uint8)|enabled:True|wants_pause:False]
    Parent objects:Nuclei
    Child objects:IdentifyG3BP1Objects
    Calculate child-parent distances?:None
    Calculate per-parent means for all child measurements?:Yes
    Calculate distances to other parents?:No
    Do you want to save the children with parents as a new object set?:Yes
    Name the output object:RelateG3BP1ToNucleusObjects
    Parent name:None

FilterObjects:[module_num:16|svn_version:'Unknown'|variable_revision_number:10|show_window:True|notes:['To compare distribution on how many cells are positive or negative for G3BP1 staining, previous identified cell objects were filtered for G3BP1.']|batch_state:array([], dtype=uint8)|enabled:True|wants_pause:False]
    Select the objects to filter:IdentifyCellObjects
    Name the output objects:G3BP1negativeCells
    Select the filtering mode:Measurements
    Select the filtering method:Limits
    Select the objects that contain the filtered objects:None
    Select the location of the rules or classifier file:Elsewhere...|
    Rules or classifier file name:rules.txt

Class number:1
Measurement count:1
Additional object count:0
Assign overlapping child to:Both parents
Keep removed objects as a separate set?:Yes
Name the objects removed by the filter:G3BP1positiveCells
Select the measurement to filter by:Children_IdentifyG3BP1Objects_Count
Filter using a minimum measurement value?:No
Minimum value:1
Filter using a maximum measurement value?:Yes
Maximum value:1.0
Allow fuzzy feature matching?:No

OverlayOutlines:[module_num:17|svn_version:'Unknown'|variable_revision_number:4|show_window:True|notes:['This module gives an overview on all identified objects and their relationsship.', 'Optional: This image can then be saved using the SaveImage module. ']|batch_state:array([], dtype=uint8)|enabled:True|wants_pause:False]
Display outlines on a blank image?:No
Select image on which to display outlines:CorrDAPI
Name the output image:OrigOverlay
Outline display mode:Color
Select method to determine brightness of outlines:Max of image
How to outline:Inner
Select outline color:#00DFDF
Select objects to display:Nuclei
Select outline color:#C94BAA
Select objects to display:G3BP1positiveCells
Select outline color:#F4F400
Select objects to display:RelateG3BP1ToCellObjects
Select outline color:white
Select objects to display:G3BP1negativeCells

MeasureObjectSizeShape:[module_num:18|svn_version:'Unknown'|variable_revision_number:3|show_window:False|notes:['To compare G3BP1 spots based on cell size, size of G3BP1 spots, cells, nucleus and cytoplasm were selected.']|batch_state:array([], dtype=uint8)|enabled:True|wants_pause:False]
    Select object sets to measure:IdentifyG3BP1Objects, RelateG3BP1ToCellObjects, RelateG3BP1ToCytoplasmObjects, RelateG3BP1ToNucleusObjects
    Calculate the Zernike features?:No
    Calculate the advanced features?:No

MeasureObjectIntensity:[module_num:19|svn_version:'Unknown'|variable_revision_number:4|show_window:False|notes:['To compare mean or max intensity of G3BP1 in cell, cytoplasm, or nuclei objects, G3BP1 as image was selected andcCytoplams, G3BP1-positive cells, and nuclei as objects.
']|batch_state:array([], dtype=uint8)|enabled:True|wants_pause:False]
    Select images to measure:G3BP1
    Select objects to measure:Cytoplasm, IdentifyCellObjects, Nuclei

MeasureObjectIntensityDistribution:[module_num:20|svn_version:'Unknown'|variable_revision_number:6|show_window:False|notes:[]|batch_state:array([], dtype=uint8)|enabled:True|wants_pause:False]
    Select images to measure:G3BP1
    Hidden:1
    Hidden:1
    Hidden:0
    Calculate intensity Zernikes?:None
    Maximum zernike moment:9
    Select objects to measure:G3BP1positiveCells
    Object to use as center?:Edges of other objects
    Select objects to use as centers:Nuclei
    Scale the bins?:Yes
    Number of bins:3
    Maximum radius:100

ExportToSpreadsheet:[module_num:21|svn_version:'Unknown'|variable_revision_number:13|show_window:True|notes:['Here, data are selected for export. ']|batch_state:array([], dtype=uint8)|enabled:True|wants_pause:False]
    Select the column delimiter:Comma (",")
    Add image metadata columns to your object data file?:Yes
    Add image file and folder names to your object data file?:No

Select the measurements to export:Yes
Calculate the per-image mean values for object measurements?:Yes
Calculate the per-image median values for object measurements?:No
Calculate the per-image standard deviation values for object measurements?:No
Output file location:Default Input Folder sub-folder|Desktop\\Submit\\Paper_supplementary\\EpitheliaCellProfiler
Create a GenePattern GCT file?:No
Select source of sample row name:Metadata
Select the image to use as the identifier:None
Select the metadata to use as the identifier:None
Export all measurement types?:Yes

measurements:RelateG3BP1ToCellObjects|AreaShape_BoundingBoxMinimum_X,RelateG3BP1ToCellObjects|AreaShape_BoundingBoxMinimum_Y,RelateG3BP1ToCellObjects|AreaShape_Eccentricity,RelateG3BP1ToCellObjects|AreaShape_Solidity,RelateG3BP1ToCellObjects|AreaShape_EulerNumber,RelateG3BP1ToCellObjects|AreaShape_ConvexArea,RelateG3BP1ToCellObjects|AreaShape_MaximumRadius,RelateG3BP1ToCellObjects|AreaShape_MinFeretDiameter,RelateG3BP1ToCellObjects|AreaShape_MeanRadius,RelateG3BP1ToCellObjects|AreaShape_Perimeter,RelateG3BP1ToCellObjects|AreaShape_Area,RelateG3BP1ToCellObjects|AreaShape_Orientation,RelateG3BP1ToCellObjects|AreaShape_BoundingBoxMaximum_Y,RelateG3BP1ToCellObjects|AreaShape_BoundingBoxMaximum_X,RelateG3BP1ToCellObjects|AreaShape_MedianRadius,RelateG3BP1ToCellObjects|AreaShape_MaxFeretDiameter,RelateG3BP1ToCellObjects|AreaShape_Compactness,RelateG3BP1ToCellObjects|AreaShape_Center_X,RelateG3BP1ToCellObjects|AreaShape_Center_Y,RelateG3BP1ToCellObjects|AreaShape_MajorAxisLength,RelateG3BP1ToCellObjects|AreaShape_BoundingBoxArea,RelateG3BP1ToCellObjects|AreaShape_MinorAxisLength,RelateG3BP1ToCellObjects|AreaShape_EquivalentDiameter,RelateG3BP1ToCellObjects|AreaShape_FormFactor,RelateG3BP1ToCellObjects|AreaShape_Extent,IdentifyG3BP1Objects|Parent_Cytoplasm,IdentifyG3BP1Objects|Parent_Nuclei,IdentifyG3BP1Objects|Parent_IdentifyCellObjects,IdentifyG3BP1Objects|AreaShape_Eccentricity,IdentifyG3BP1Objects|AreaShape_MaximumRadius,IdentifyG3BP1Objects|AreaShape_MaxFeretDiameter,IdentifyG3BP1Objects|AreaShape_Area,IdentifyG3BP1Objects|AreaShape_BoundingBoxMaximum_X,IdentifyG3BP1Objects|AreaShape_BoundingBoxMaximum_Y,IdentifyG3BP1Objects|AreaShape_MinFeretDiameter,IdentifyG3BP1Objects|AreaShape_MajorAxisLength,IdentifyG3BP1Objects|AreaShape_MinorAxisLength,IdentifyG3BP1Objects|AreaShape_Compactness,IdentifyG3BP1Objects|AreaShape_Center_X,IdentifyG3BP1Objects|AreaShape_Center_Y,IdentifyG3BP1Objects|AreaShape_Solidity,IdentifyG3BP1Objects|AreaShape_MeanRadius,IdentifyG3BP1Objects|AreaShape_BoundingBoxArea,IdentifyG3BP1Objects|AreaShape_FormFactor,IdentifyG3BP1Objects|AreaShape_Orientation,IdentifyG3BP1Objects|AreaShape_EquivalentDiameter,IdentifyG3BP1Objects|AreaShape_BoundingBoxMinimum_Y,IdentifyG3BP1Objects|AreaShape_BoundingBoxMinimum_X,IdentifyG3BP1Objects|AreaShape_MedianRadius,IdentifyG3BP1Objects|AreaShape_Extent,IdentifyG3BP1Objects|AreaShape_EulerNumber,IdentifyG3BP1Objects|AreaShape_Perimeter,IdentifyG3BP1Objects|AreaShape_ConvexArea,IdentifyG3BP1Objects|Children_RelateG3BP1ToCellObjects_Count,IdentifyG3BP1Objects|Children_RelateG3BP1ToCytoplasmObjects_Count,IdentifyG3BP1Objects|Children_RelateG3BP1ToNucleusObjects_Count,IdentifyG3BP1Objects|Number_Object_Number,RelateG3BP1ToNucleusObjects|AreaShape_FormFactor,RelateG3BP1ToNucleusObjects|AreaShape_MaxFeretDiameter,RelateG3BP1ToNucleusObjects|AreaShape_MajorAxisLength,RelateG3BP1ToNucleusObjects|AreaShape_EquivalentDiameter,RelateG3BP1ToNucleusObjects|AreaShape_Eccentricity,RelateG3BP1ToNucleusObjects|AreaShape_BoundingBoxMinimum_Y,RelateG3BP1ToNucleusObjects|AreaShape_BoundingBoxMinimum_X,RelateG3BP1ToNucleusObjects|AreaShape_MaximumRadius,RelateG3BP1ToNucleusObjects|AreaShape_BoundingBoxMaximum_Y,RelateG3BP1ToNucleusObjects|AreaShape_BoundingBoxMaximum_X,RelateG3BP1ToNucleusObjects|AreaShape_Compactness,RelateG3BP1ToNucleusObjects|AreaShape_MinFeretDiameter,RelateG3BP1ToNucleusObjects|AreaShape_Extent,RelateG3BP1ToNucleusObjects|AreaShape_ConvexArea,RelateG3BP1ToNucleusObjects|AreaShape_Perimeter,RelateG3BP1ToNucleusObjects|AreaShape_BoundingBoxArea,RelateG3BP1ToNucleusObjects|AreaShape_Area,RelateG3BP1ToNucleusObjects|AreaShape_Center_Y,RelateG3BP1ToNucleusObjects|AreaShape_Center_X,RelateG3BP1ToNucleusObjects|AreaShape_MedianRadius,RelateG3BP1ToNucleusObjects|AreaShape_EulerNumber,RelateG3BP1ToNucleusObjects|AreaShape_MeanRadius,RelateG3BP1To

Representation of Nan/Inf:Null

Add a prefix to file names?:Yes

Filename prefix:25032025

Overwrite existing files without warning?:Yes

Data to export:Do not use
Combine these object measurements with those of the previous object?:No
File name:DATA.csv
Use the object name for the file name?:Yes

```
Key,Value
CellProfiler_Version,4.2.7
ChannelType_DAPI,Grayscale
ChannelType_EpCAM,Grayscale
ChannelType_G3BP1,Grayscale
Metadata_Tags,"[""ImageNumber""]"
Pipeline_Pipeline,"CellProfiler Pipeline: http://www.cellprofiler.org
Version:5
DateRevision:427
GitHash:
ModuleCount:20
HasImagePlaneDetails:False

Images:[module_num:1|svn_version:'Unknown'|variable_revision_number:2|show_window:False|notes:
['To begin creating your project, use the Images module to compile a list of files and/or folders that you
want to analyze. You can also specify a set of rules to include only the desired files in your selected
folders.', '', '']|batch_state:array([], dtype=uint8)|enabled:True|wants_pause:False]
    :
    Filter images?:Custom
    Select the rule criteria:or (extension does isimage) (file does contain ""overlay"")

Metadata:[module_num:2|svn_version:'Unknown'|variable_revision_number:6|show_window:False|not
es:['The Metadata module optionally allows you to extract information describing your images (i.e,
metadata) which will be stored along with your measurements. This information can be contained in the
file name and/or location, or in an external file.']|batch_state:array([],
    Extract metadata?:No
    Metadata data type:Text
    Metadata types:{}
    Extraction method count:1
    Metadata extraction method:Extract from file/folder names
    Metadata source:File name
    Regular expression to extract from file name:^(?P<Plate>.*)_(?P<Well>[A-P][0-9]{2})_s(?P<Site>[0-
9])_w(?P<ChannelNumber>[0-9])
    Regular expression to extract from folder name:(?P<Date>[0-9]{4}_[0-9]{2}_[0-9]{2})$
    Extract metadata from:Images matching a rule
    Select the filtering criteria:and (file does contain ""5103"")
    Metadata file location:Elsewhere...|
    Match file and image metadata:[]
    Use case insensitive matching?:No
    Metadata file name:None
    Does cached metadata exist?:No

NamesAndTypes:[module_num:3|svn_version:'Unknown'|variable_revision_number:8|show_window:Fal
se|notes:['The NamesAndTypes module allows you to assign a meaningful name to each image by which
other modules will refer to it.', '', '']|batch_state:array([], dtype=uint8)|enabled:True|wants_pause:False]
    Assign a name to:Images matching rules
    Select the image type:Color image
    Name to assign these images:Image
    Match metadata:[]
    Image set matching method:Order
```

Set intensity range from:Image metadata
Assignments count:3
Single images count:0
Maximum intensity:255.0
Process as 3D?:No
Relative pixel spacing in X:1.0
Relative pixel spacing in Y:1.0
Relative pixel spacing in Z:1.0
Select the rule criteria:and (file does containregexp ""ch00"")
Name to assign these images:EpCAM
Name to assign these objects:Cell
Select the image type:Grayscale image
Set intensity range from:Image metadata
Maximum intensity:255.0
Select the rule criteria:and (file does containregexp ""ch01"")
Name to assign these images:DAPI
Name to assign these objects:Cell
Select the image type:Grayscale image
Set intensity range from:Image metadata
Maximum intensity:255.0
Select the rule criteria:and (file does containregexp ""ch02"")
Name to assign these images:G3BP1
Name to assign these objects:Cell
Select the image type:Grayscale image
Set intensity range from:Image metadata
Maximum intensity:255.0

Groups:[module_num:4|svn_version:'Unknown'|variable_revision_number:2|show_window:False|notes:['The Groups module optionally allows you to split your list of images into image subsets (groups) which will be processed independently of each other. Examples of groupings include screening batches, microtiter plates, time-lapse movies, etc.']|batch_state:array([],
Do you want to group your images?:No
grouping metadata count:1
Metadata category:None

CorrectIlluminationApply:[module_num:5|svn_version:'Unknown'|variable_revision_number:5|show_window:False|notes:['Accurate segmentation of nucleus objects is crucial for subsequent steps and is highly dependent on the effectiveness of the staining process. Substracting EpCAM signals from the DAPI channel aids in the segmentation of IEC and reduced the EpCAM signal that is mistakenely present in the lamina propria. This signal can be filtered out more easily in the later treshold settings additionally. ']|batch_state:array([], dtype=uint8)|enabled:True|wants_pause:False]
Select the input image:DAPI
Name the output image:CorrBlue
Select the illumination function:EpCAM
Select how the illumination function is applied:Subtract
Set output image values less than 0 equal to 0?:Yes
Set output image values greater than 1 equal to 1?:Yes

IdentifyPrimaryObjects:[module_num:6|svn_version:'Unknown'|variable_revision_number:15|show_window:True|notes:['Since potential immune cells and epithelial cells differ in size and morphology, it was necessary to seperate them into 2 different pipelines (LaminaCellProfiler and EpitheliaCellProfiler). Therefore, during segmentation of immune cell nuclei, objects in the intestinal area were not considered as the differences, as mentioned previolusy, were too significant. Objects inside the intestinal region are filtered out in the subsequent steps anyway. ', '', 'Over- or under segmentation is common, particulary in complex tissues. To minimize this, it is recommended to acquire high-quality images. However, it should be noted that approx. 10% error rate per image can be expected. ']|batch_state:array([], dtype=uint8)|enabled:True|wants_pause:False]
    Select the input image:CorrBlue
    Name the primary objects to be identified:IdentifyNucleiObjects
    Typical diameter of objects, in pixel units (Min,Max):12,70
    Discard objects outside the diameter range?:Yes
    Discard objects touching the border of the image?:Yes
    Method to distinguish clumped objects:Shape
    Method to draw dividing lines between clumped objects:Shape
    Size of smoothing filter:10
    Suppress local maxima that are closer than this minimum allowed distance:10
    Speed up by using lower-resolution image to find local maxima?:Yes
    Fill holes in identified objects?:After both thresholding and declumping
    Automatically calculate size of smoothing filter for declumping?:No
    Automatically calculate minimum allowed distance between local maxima?:No
    Handling of objects if excessive number of objects identified:Continue
    Maximum number of objects:500
    Use advanced settings?:Yes
    Threshold setting version:12
    Threshold strategy:Global
    Thresholding method:Minimum Cross-Entropy
    Threshold smoothing scale:2
    Threshold correction factor:1.0
    Lower and upper bounds on threshold:0.12,1.0
    Manual threshold:0.0
    Select the measurement to threshold with:None
    Two-class or three-class thresholding?:Two classes
    Log transform before thresholding?:No
    Assign pixels in the middle intensity class to the foreground or the background?:Foreground
    Size of adaptive window:25
    Lower outlier fraction:0.05
    Upper outlier fraction:0.05
    Averaging method:Mean
    Variance method:Standard deviation
    # of deviations:2.0
    Thresholding method:Minimum Cross-Entropy

IdentifyPrimaryObjects:[module_num:7|svn_version:'Unknown'|variable_revision_number:15|show_window:False|notes:['Here, we define the intestinal region as an object based on the green (EpCAM) channel. ']|batch_state:array([], dtype=uint8)|enabled:True|wants_pause:False]
    Select the input image:EpCAM
    Name the primary objects to be identified:IdentifyIntestine
    Typical diameter of objects, in pixel units (Min,Max):50,900

Discard objects outside the diameter range?:Yes
Discard objects touching the border of the image?:No
Method to distinguish clumped objects:None
Method to draw dividing lines between clumped objects:Intensity
Size of smoothing filter:10
Suppress local maxima that are closer than this minimum allowed distance:100
Speed up by using lower-resolution image to find local maxima?:Yes
Fill holes in identified objects?:After declumping only
Automatically calculate size of smoothing filter for declumping?:Yes
Automatically calculate minimum allowed distance between local maxima?:Yes
Handling of objects if excessive number of objects identified:Continue
Maximum number of objects:500
Use advanced settings?:Yes
Threshold setting version:12
Threshold strategy:Global
Thresholding method:Minimum Cross-Entropy
Threshold smoothing scale:1.3488
Threshold correction factor:1.0
Lower and upper bounds on threshold:0.02,1.0
Manual threshold:0.0
Select the measurement to threshold with:None
Two-class or three-class thresholding?:Two classes
Log transform before thresholding?:No
Assign pixels in the middle intensity class to the foreground or the background?:Foreground
Size of adaptive window:50
Lower outlier fraction:0.05
Upper outlier fraction:0.05
Averaging method:Mean
Variance method:Standard deviation
# of deviations:2.0
Thresholding method:Minimum Cross-Entropy

ExpandOrShrinkObjects:[module_num:8|svn_version:'Unknown'|variable_revision_number:2|show_window:False|notes:['Sometimes cells close to intestinal crypts overlap within EpCAM staining. By shrinking or expanding the EpCAM objects by few pixels, these cells can be kept or removed']|batch_state:array([], dtype=uint8)|enabled:True|wants_pause:False]
Select the input objects:IdentifyIntestine
Name the output objects:ShrunkenIntestine
Select the operation:Shrink objects by a specified number of pixels
Number of pixels by which to expand or shrink:1
Fill holes in objects so that all objects shrink to a single point?:Yes
Expand or shrink measurement:None

MaskObjects:[module_num:9|svn_version:'Unknown'|variable_revision_number:3|show_window:True|notes:['In this module, all DAPI-identified objects that can be found within the EpCAM-stained Mask are removed. ']|batch_state:array([], dtype=uint8)|enabled:True|wants_pause:False]
Select objects to be masked:IdentifyNucleiObjects
Name the masked objects:MaskedNuclei
Mask using a region defined by other objects or by binary image?:Objects
Select the masking object:ShrunkenIntestine

Select the masking image:None
Handling of objects that are partially masked:Remove
Fraction of object that must overlap:0.5
Numbering of resulting objects:Renumber
Invert the mask?:Yes

MeasureObjectSizeShape:[module_num:10|svn_version:'Unknown'|variable_revision_number:3|show_window:False|notes:['Size and nuclei form is measured in this step to filter out excessive cells (due to oversegmentation) and potentially myofibroblasts.']|batch_state:array([], dtype=uint8)|enabled:True|wants_pause:False]
Select object sets to measure:MaskedNuclei
Calculate the Zernike features?:No
Calculate the advanced features?:No

FilterObjects:[module_num:11|svn_version:'Unknown'|variable_revision_number:10|show_window:False|notes:['Myofibroblasts are contractile cells with a central role in the remodeling and the restoration of damaged gastrointestinal tissue. These subepithelial intestinal fibroblasts are present within the lamina propria and often surround the crypt epithelium. Immunologic makers exist to aid in the identification of these cellls, but none is absolutely specific. However, using ""MeasureObjectSizeShape"" myofibroblasts within the lamina propria can be filtered and seperataed from immune cells due to their characteristic shape and an eccentricity value over 0.9 if not sufficient seperated alreay in the previos masking step. ', 'Also, by filtering cells by their maximal area, we filtered out obvious oversegmented cells (two or three cells that were recognized as one). ']|batch_state:array([],
Select the objects to filter:MaskedNuclei
Name the output objects:Nuclei
Select the filtering mode:Measurements
Select the filtering method:Limits
Select the objects that contain the filtered objects:None
Select the location of the rules or classifier file:Elsewhere...|
Rules or classifier file name:rules.txt
Class number:1
Measurement count:2
Additional object count:0
Assign overlapping child to:Both parents
Keep removed objects as a separate set?:Yes
Name the objects removed by the filter:FilteredCells
Select the measurement to filter by:AreaShape_Eccentricity
Filter using a minimum measurement value?:No
Minimum value:0.0
Filter using a maximum measurement value?:Yes
Maximum value:0.97
Select the measurement to filter by:AreaShape_Area
Filter using a minimum measurement value?:No
Minimum value:0.0
Filter using a maximum measurement value?:Yes
Maximum value:1600
Allow fuzzy feature matching?:No

IdentifySecondaryObjects:[module_num:12|svn_version:'Unknown'|variable_revision_number:10|show_window:False|notes:['Here, cells outer edges are identified. In this study, no additional cell marker was used for immune cells, therefore, the Distance-N method was used to assign an outer edge, based on pixels that expand from the nucleus objects. ']|batch_state:array([],
Select the input objects:Nuclei
Name the objects to be identified:IdentifyCellObjects
Select the method to identify the secondary objects:Distance - N
Select the input image:CorrBlue
Number of pixels by which to expand the primary objects:7
Regularization factor:0.05
Discard secondary objects touching the border of the image?:No
Discard the associated primary objects?:No
Name the new primary objects:FilteredNuclei
Fill holes in identified objects?:Yes
Threshold setting version:12
Threshold strategy:Global
Thresholding method:Minimum Cross-Entropy
Threshold smoothing scale:0.0
Threshold correction factor:1.0
Lower and upper bounds on threshold:0.0,1.0
Manual threshold:0.0
Select the measurement to threshold with:None
Two-class or three-class thresholding?:Two classes
Log transform before thresholding?:No
Assign pixels in the middle intensity class to the foreground or the background?:Foreground
Size of adaptive window:50
Lower outlier fraction:0.05
Upper outlier fraction:0.05
Averaging method:Mean
Variance method:Standard deviation
# of deviations:2.0
Thresholding method:Minimum Cross-Entropy

EnhanceOrSuppressFeatures:[module_num:13|svn_version:'Unknown'|variable_revision_number:7|show_window:False|notes:['Enhancing features such as speckles can increase the contrast between the objects of interest and the backgroud, making it easier to distinguish specific features within the image. Also, algorithms can detect more accurately, segment, and classify objects. ']|batch_state:array([], dtype=uint8)|enabled:True|wants_pause:False]
Select the input image:G3BP1
Name the output image:EnhanceOrSuppressFeatures
Select the operation:Enhance
Feature size:10
Feature type:Speckles
Range of hole sizes:1,10
Smoothing scale:2.0
Shear angle:0.0
Decay:0.95
Enhancement method:Tubeness
Speed and accuracy:Fast
Rescale result image:No

IdentifyPrimaryObjects:[module_num:14|svn_version:'Unknown'|variable_revision_number:15|show_window:False|notes:['Here, we identify G3BP1 granules in the red (G3BP1) channel as object to related them in the next steps.']|batch_state:array([], dtype=uint8)|enabled:True|wants_pause:False]
    Select the input image:EnhanceOrSuppressFeatures
    Name the primary objects to be identified:IdentifySpotObjects
    Typical diameter of objects, in pixel units (Min,Max):2,15
    Discard objects outside the diameter range?:Yes
    Discard objects touching the border of the image?:Yes
    Method to distinguish clumped objects:Intensity
    Method to draw dividing lines between clumped objects:Intensity
    Size of smoothing filter:10
    Suppress local maxima that are closer than this minimum allowed distance:7.0
    Speed up by using lower-resolution image to find local maxima?:Yes
    Fill holes in identified objects?:After both thresholding and declumping
    Automatically calculate size of smoothing filter for declumping?:Yes
    Automatically calculate minimum allowed distance between local maxima?:Yes
    Handling of objects if excessive number of objects identified:Continue
    Maximum number of objects:500
    Use advanced settings?:Yes
    Threshold setting version:12
    Threshold strategy:Global
    Thresholding method:Otsu
    Threshold smoothing scale:2
    Threshold correction factor:1.0
    Lower and upper bounds on threshold:0.04,1.0
    Manual threshold:0.0
    Select the measurement to threshold with:None
    Two-class or three-class thresholding?:Two classes
    Log transform before thresholding?:No
    Assign pixels in the middle intensity class to the foreground or the background?:Foreground
    Size of adaptive window:50
    Lower outlier fraction:0.05
    Upper outlier fraction:0.05
    Averaging method:Mean
    Variance method:Standard deviation
    # of deviations:2.0
    Thresholding method:Sauvola

RelateObjects:[module_num:15|svn_version:'Unknown'|variable_revision_number:5|show_window:False|notes:['Here, we relate previous identifiy G3BP1 spots with cells, cytoplasm or nucleus (in seperate steps) to compare intensity, respectively. ', '', 'Note: The output later is extensive, so take the time to labbel objects appropriate, to retrieve desired measurements. ']|batch_state:array([], dtype=uint8)|enabled:True|wants_pause:False]
    Parent objects:IdentifyCellObjects
    Child objects:IdentifySpotObjects
    Calculate child-parent distances?:None
    Calculate per-parent means for all child measurements?:Yes
    Calculate distances to other parents?:No
    Do you want to save the children with parents as a new object set?:Yes

    Name the output object:RelateG3BP1ToCellObjects
    Parent name:None

FilterObjects:[module_num:16|svn_version:'Unknown'|variable_revision_number:10|show_window:False|notes:['To compare distribution on how many cells are positive or negative for G3BP1 staining, previous identified cell objects were filtered for G3BP1.']|batch_state:array([], dtype=uint8)|enabled:True|wants_pause:False]
    Select the objects to filter:IdentifyCellObjects
    Name the output objects:G3BP1negativeCells
    Select the filtering mode:Measurements
    Select the filtering method:Limits
    Select the objects that contain the filtered objects:None
    Select the location of the rules or classifier file:Elsewhere...|
    Rules or classifier file name:rules.txt
    Class number:1
    Measurement count:1
    Additional object count:0
    Assign overlapping child to:Both parents
    Keep removed objects as a separate set?:Yes
    Name the objects removed by the filter:G3BP1positive
    Select the measurement to filter by:Children_IdentifySpotObjects_Count
    Filter using a minimum measurement value?:No
    Minimum value:1
    Filter using a maximum measurement value?:Yes
    Maximum value:1.0
    Allow fuzzy feature matching?:No

OverlayOutlines:[module_num:17|svn_version:'Unknown'|variable_revision_number:4|show_window:True|notes:['This module gives an overview on all identified objects and their relationsship.', 'Optional: This image can then be saved using the SaveImage module. ']|batch_state:array([], dtype=uint8)|enabled:True|wants_pause:False]
    Display outlines on a blank image?:No
    Select image on which to display outlines:DAPI
    Name the output image:OrigOverlay1
    Outline display mode:Color
    Select method to determine brightness of outlines:Max of image
    How to outline:Thick
    Select outline color:#00CACA
    Select objects to display:Nuclei
    Select outline color:yellow
    Select objects to display:RelateG3BP1ToCellObjects
    Select outline color:white
    Select objects to display:G3BP1negativeCells
    Select outline color:#CA0065
    Select objects to display:G3BP1positive

MeasureObjectIntensity:[module_num:18|svn_version:'Unknown'|variable_revision_number:4|show_window:False|notes:['Here we used MeasureOjectIntensity to measure pixel intensity of G3BP1 in G3BP1+ positive cells of healthy control and non-IBD']|batch_state:array([], dtype=uint8)|enabled:True|wants_pause:False]

Select images to measure:G3BP1
Select objects to measure:G3BP1positive, IdentifyCellObjects, IdentifySpotObjects

MeasureObjectSizeShape:[module_num:19|svn_version:'Unknown'|variable_revision_number:3|show_window:False|notes:['This module was used, to compare G3BP1 spot size and nuclei morphology between healthy control and ulcerative colitis patients in remission. ']|batch_state:array([], dtype=uint8)|enabled:True|wants_pause:False]
Select object sets to measure:IdentifySpotObjects, Nuclei
Calculate the Zernike features?:No
Calculate the advanced features?:No

ExportToSpreadsheet:[module_num:20|svn_version:'Unknown'|variable_revision_number:13|show_window:False|notes:['Here, data are selected for export. ']|batch_state:array([], dtype=uint8)|enabled:True|wants_pause:False]
Select the column delimiter:Comma ("","")
Add image metadata columns to your object data file?:No
Add image file and folder names to your object data file?:No
Select the measurements to export:Yes
Calculate the per-image mean values for object measurements?:No
Calculate the per-image median values for object measurements?:No
Calculate the per-image standard deviation values for object measurements?:No
Output file location:Default Input Folder sub-folder|Desktop\\Submit\\Paper_supplementary\\PropiCellProfiler
Create a GenePattern GCT file?:No
Select source of sample row name:Metadata
Select the image to use as the identifier:None
Select the metadata to use as the identifier:None
Export all measurement types?:Yes
Press button to select measurements:Experiment|Run_Timestamp,Experiment|Modification_Timestamp,Experiment|Pipeline_Pipeline,Experiment|CellProfiler_Version
Representation of Nan/Inf:Null
Add a prefix to file names?:Yes
Filename prefix:PropiCellProfiler_Output
Overwrite existing files without warning?:Yes
Data to export:Do not use
Combine these object measurements with those of the previous object?:No
File name:DATA.csv
Use the object name for the file name?:Yes
